# Two independent allohexaploidizations and genomic fractionation in Solanales

**DOI:** 10.3389/fpls.2022.1001402

**Published:** 2022-09-23

**Authors:** Yan Zhang, Lan Zhang, Qimeng Xiao, Chunyang Wu, Jiaqi Zhang, Qiang Xu, Zijian Yu, Shoutong Bao, Jianyu Wang, Yu Li, Li Wang, Jinpeng Wang

**Affiliations:** ^1^Center for Genomics and Computational Biology, School of Life Sciences, North China University of Science and Technology, Tangshan, Hebei, China; ^2^University of Chinese Academy of Sciences, Beijing, China; ^3^State Key Laboratory of Systematic and Evolutionary Botany, Institute of Botany, Chinese Academy of Sciences, Beijing, China

**Keywords:** Solanaceae, Convolvulaceae, polyploidization, genomic fractionation, chromosomal structural variation, BMY genes\keywordbelowspace-30pt

## Abstract

Solanales, an order of flowering plants, contains the most economically important vegetables among all plant orders. To date, many Solanales genomes have been sequenced. However, the evolutionary processes of polyploidization events in Solanales and the impact of polyploidy on species diversity remain poorly understood. We compared two representative Solanales genomes (*Solanum lycopersicum* L. and *Ipomoea triloba* L.) and the *Vitis vinifera* L. genome and confirmed two independent polyploidization events. Solanaceae common hexaploidization (SCH) and Convolvulaceae common hexaploidization (CCH) occurred ∼43–49 and ∼40–46 million years ago (Mya), respectively. Moreover, we identified homologous genes related to polyploidization and speciation and constructed multiple genomic alignments with *V. vinifera* genome, providing a genomic homology framework for future Solanales research. Notably, the three polyploidization-produced subgenomes in both *S. lycopersicum* and *I. triloba* showed significant genomic fractionation bias, suggesting the allohexaploid nature of the SCH and CCH events. However, we found that the higher genomic fractionation bias of polyploidization-produced subgenomes in Solanaceae was likely responsible for their more abundant species diversity than that in Convolvulaceae. Furthermore, through genomic fractionation and chromosomal structural variation comparisons, we revealed the allohexaploid natures of SCH and CCH, both of which were formed by two-step duplications. In addition, we found that the second step of two paleohexaploidization events promoted the expansion and diversity of β-amylase (BMY) genes in Solanales. These current efforts provide a solid foundation for future genomic and functional exploration of Solanales.

## Introduction

The order Solanales is the third largest group of economically important crops in the world, with more than 4,459 species belonging to 186 genera. Solanales contains abundant vegetable crops, such as *Solanum lycopersicum* L. (2*n* = 2x = 24), *Ipomoea triloba* L. (2*n* = 2x = 30), and *Solanum tuberosum* L. (2*n* = 2x = 48), as well as medicinal plants, such as *Lycium chinense* Miller (2*n* = 2x = 24) (Sato et al., 2012). Because of their high economic value, the genome sequences of 40 species have been obtained in the two largest families, Solanaceae and Convolvulaceae. Solanaceae ranks third among plant families in economic importance, containing more than 95 genera and 2,300 species (Cao et al., 2021; Robertson et al., 2011). *S. lycopersicum*, a representative Solanaceae species, is the second most important economic vegetable crop in the world (Hammond, 2017) and is rich in vitamins, minerals, and fiber, providing an important model system for fruit development (Sato et al., 2012; Suzuki et al., 2015; Takei et al., 2021). In addition, Convolvulaceae has more than 99 genera and 2,700 species (Poczai et al., 2022). *I. triloba*, as a representative Convolvulaceae species, is a diploid wild relative of cultivated *Ipomoea batatas* L., which is one of two food crops in Convolvulaceae and the fourth most significant economic crop in China (Yang et al., 2017). *I. triloba* plays a key role in the constitution of the cultivated *I. batatas* genome and provides an opportunity to study the genetic improvement of cultivated *I. batatas* germplasm (Wang et al., 2019b). The study of *I. triloba* can provide a foundation for revealing the diversity of Convolvulaceae genomes under natural conditions. In addition, the *Vitis vinifera* L. genome (2*n* = 2x = 38), a core eudicot, has been sequenced, revealing that it has fewer chromosomal rearrangements than most other eudicots (Jaillon et al., 2007).

Polyploidization refers to the duplication of all chromosomes of a species, also known as whole-genome duplication (WGD), which has been widespread throughout the evolutionary history of green plants (Viridiplantae) (Frawley and Orr-Weaver, 2015; Soltis et al., 2015; Aguirre et al., 2019). Following the polyploidy, the large number of chromosomal rearrangements and genomic fractionation have increased the complexity of the plant genomes (Paterson et al., 2004; Wang et al., 2005; Murat et al., 2017; Yang et al., 2017; Wang et al., 2018a; Zhuang et al., 2019). This has led to great challenges in determining the ploidy of the ancient polyploid ancestors and the timing of the occurrence of polyploidization events. There is no lucky escape from this difficulty in the study of genome polyploidy evolution in Solanales plants. In a previous study of *S. tuberosum* genome, the researchers reported that there were a tetraploidization event in family Solanaceae after the core eudicot common hexaploidization (ECH) event, and suggested this WGD event was likely shared by many Solanaceae plants (Xu et al., 2011). However, some recent studies prefer to identify the ancient WGD event in Solanaceae as a common hexaploidization event (SCH) (Xu et al., 2011; Barchi et al., 2019; Cao et al., 2021). In addition, previous studies have also inferred that the SCH event occurred at different times. For example, in the study of *S. tuberosum* genome, the occurrence time of SCH was ∼67 million years ago (Mya) (Xu et al., 2011), but in the study of *Lycium chinense* genome, it was dated as 69 Mya (Cao et al., 2021). Another family of Solanales, Convolvulaceae, has also reported an ancient hexaploidization event [named Convolvulaceae common hexaploidization (CCH) event], but the time of occurrence has not been determined. In the study of *Ipomoea nil* (Linnaeus) Roth and *Cuscuta australis* R. genomes, researchers reported that the CCH event occurred ∼70 Mya, while the research on *I. batatas* points to a different scenario, in which the CCH event occurred before ∼46 Mya (Wu et al., 2018). At present, although we have a certain understanding of the history of the polyploidy of Solanales, the times of their polyploidization events are still unclear, and even we cannot determine whether the Solanaceae and Convolvulaceae shared a hexaploidization in Solanales.

Generally, polyploidization generates two forms of polyploidy: autopolyploidy and allopolyploidy. In allopolyploidy, genomic fractionation bias is characterized by extensive chromosomal rearrangements and massive gene loss after polyploidization events, while this phenomenon does not exist in autopolyploidy (Lyons et al., 2008; Wang et al., 2011). In addition, allopolyploids have heterosis and higher offspring survival rates and may contribute to the establishment of large families, such as Solanaceae, Poaceae, Fabaceae, and Brassicaceae (Group et al., 2001; Magallón and Sanderson, 2001; Young and Bharti, 2012; Wang et al., 2019a). In the study of the *Brassica rapa* L. and *Lupinus albus* L. genomes, researchers proposed that their paleo hexaploidization event was formed by two-step duplications, that is, the two subgenomes with more fractionation came together first, and then the subgenomes with less fractionation joined and finally formed a triploid (Wang et al., 2011). Although Solanaceae and Convolvulaceae are both large families with allopolyploids, their polyploidy formation process has not been elucidated. In recent research, a method was proposed to determine the phylogenetic evolutionary relationships of angiosperms by identifying the variation in their genome structures (Qin et al., 2021), which could help to resolve evolutionary events that occurred in a very compressed evolutionary window. Exploring genome structural variation to decipher the polyploidization evolutionary process of Solanales may provide new insights into their genomic evolution.

Here, by using the previously proposed pipeline (Wang et al., 2018a), we compared two representative Solanales genomes (*S. lycopersicum* and *I. triloba*) and a reference genome (*V. vinifera*). We identified the genomic homologies associated with key evolutionary events of polyploidization and species divergence in Solanales, and redated the times of the polyploidizations in Solanaceae and Convolvulaceae. By performing genomic fractionation and structural variation comparisons, we explored the genome duplication models of polyploidization events in two families of Solanales. Besides, the function of β-amylase (BMY) is to break down starch for use in grain germination, seedling growth, endosperm development, and response to abiotic stress (Srivastava and Kayastha, 2014). It is worth noting that recent studies have shown that the BMY gene also plays an important role in the formation of storage stems. Therefore, based on the importance of underground storage roots to some Solanales, such as *I. batatas* (Wang et al., 2019b), we identified the BMY members in the studied genomes, and explored whether polyploidizations and biased genomic differentiation promoted the expansion and diversity of the BMY family in Solanales.

## Results

### Syntenic genes and *Ks* distribution characteristics

To revisit the polyploidization events of Solanaceae and Convolvulaceae, with *V. vinifera* as an excellent reference genome, we compared the genomic synteny within and between the *S. lycopersicum* and *I. triloba* genomes. This study identified 7,175 syntenic gene pairs located in 711 blocks of the *V. vinifera* genome, each containing at least four syntenic gene pairs (Supplementary Table 1). More syntenic genes were found in the genomes of *S. lycopersicum* and *I. triloba*, mainly because both genomes were affected by an additional WGD event after ECH; there were 17,006 and 28,222 syntenic gene pairs located in 1,916 and 3,113 blocks for *S. lycopersicum* and *I. triloba*, respectively (Supplementary Table 1). In addition, we compared the genomic synteny among the *V. vinifera*, *S. lycopersicum*, and *I. triloba* genomes (Supplementary Table 1). A larger number of syntenic gene pairs per block, with more than 50 syntenic gene pairs, were found between *V. vinifera* and *I. triloba* compared to those between *V. vinifera* and *S. lycopersicum*. Analysis revealed 6,852 and 5,910 syntenic gene pairs located in 82 and 61 blocks between *V. vinifera* and the genomes of *I. triloba* and *S. lycopersicum*, respectively (Supplementary Table 1), showing that *I. triloba* had better homology with *V. vinifera*.

**FIGURE 1:**
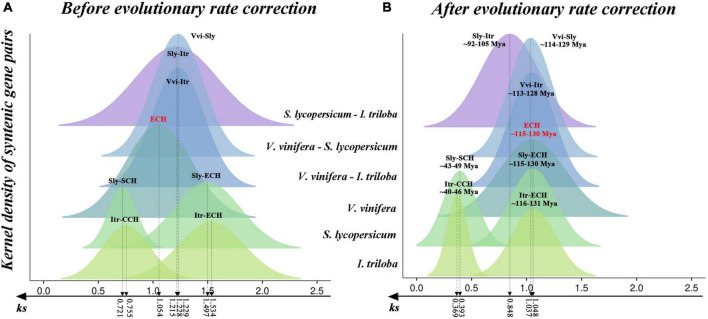
Original and corrected *Ks* distribution among syntenic genes within and between genomes of *Solanum lycopersicum* (Sly), *Ipomoea triloba* (Itr), and *Vitis vinifera* (Vvi). **(A)** Distribution of *Ks* values between syntenic blocks before correction. **(B)** Distribution of *Ks* values among syntenic blocks after evolutionary rate correction. The different colored peaks indicate the normal distribution of the *Ks* values of syntenic blocks.

The divergence degree in *Ks* of each syntenic block was analyzed, and the *Ks* peaks associated with polyploidization and speciation events among considered species were inferred (Figure 1A and Supplementary Table 2). Of these, the *Ks* distribution of syntenic blocks in *V. vinifera* showed a clear unimodal pattern with a peak at ∼1.054, corresponding to the ECH event. In addition, a clear bimodal *Ks* distribution pattern (one large and one small) was detected in the *S. lycopersicum* and *I. triloba* genomes (Figure 1A). The larger values of ∼1.497 and ∼1.534 were related to the ECH event, and the smaller values of ∼0.721 and ∼0.755 corresponded to further WGD events in *S. lycopersicum* and *I. triloba*, respectively. Moreover, we identified the *Ks* peaks of syntenic blocks that corresponded to species divergence (Figure 1A and Supplementary Table 2). The three peaks between *V. vinifera* and *S. lycopersicum*; *V. vinifera* and *I. triloba*; and *S. lycopersicum* and *I. triloba* were located at ∼1.215, 1.229, and 1.230, respectively. The estimation of these *Ks* peaks associated with evolutionary events could facilitate the identification of paralogous and orthologous genomic regions and further serve as homologous evidence to infer the phylogenetic placements of WGD events.

### Two independent hexaploidization events in Solanales

**FIGURE 2:**
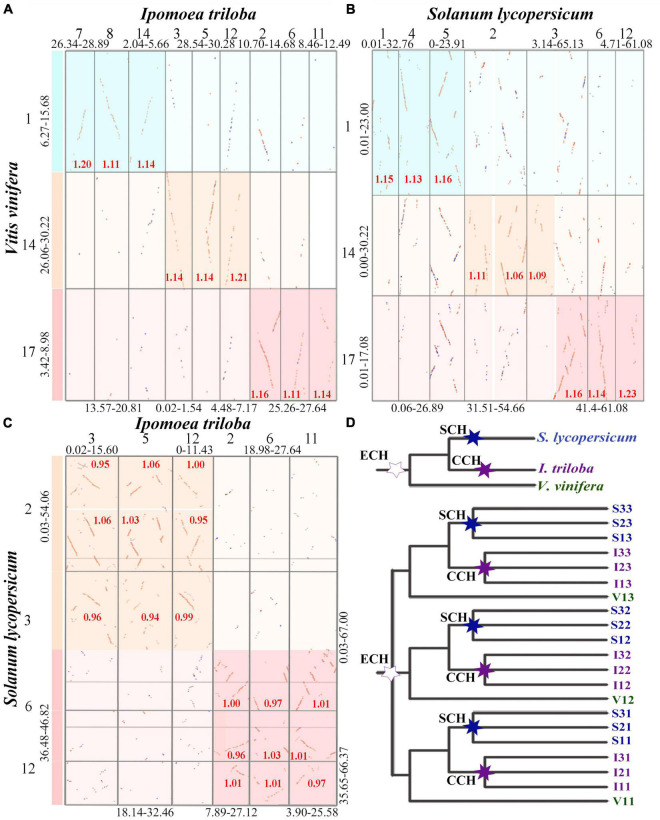
Inference of the polyploidization events for *Solanum lycopersicum* and *Ipomoea triloba*. In genomic synteny local dotplots, the dark highlight boxes indicate orthologous genomic regions identified between compared genomes, and the light boxes indicate identified outparalogous genomic regions. The *Ks* median of gene pairs in homologous chromosomal regions is marked in the boxes. The lengths of compared chromosomes from each genome are shown in Mb. The en dash “-” indicates a range. **(A)** Local synteny block dotplot between the genomes of *V. vinifera* and *I. triloba*; **(B)** local synteny block dotplot between the genomes of *Vitis vinifera* and *I. triloba*; **(C)** local synteny block dotplot between the genomes of *S. lycopersicum* and *I. triloba*; **(D)** species and inferred homologous gene tree of *V. vinifera* (Vvi), *I. triloba* (Itr), and *S. lycopersicum* (Sly). The hexagonal star labels on the trees indicate the inferred polyploidizations among three considered species, in which the Solanaceae common hexaploidization (SCH) is color-coded blue, the Convolvulaceae common hexaploidization (CCH) is color-coded purple, and the core eudicot common hexaploidization (ECH) is color-coded dark green.

According to the median *Ks* of syntenic blocks in homologous chromosomal regions, we further analyzed the homoeologous regions within and between the studied genomes produced by polyploidization and species divergence. It was found that each of nine orthologous chromosomal regions in *I. triloba* corresponded to three paralogous chromosomal regions of *V. vinifera* generated from ECH. For example, the three paralogous chromosomal regions Vvi1, Vvi14, and Vvi17 generated from ECH in *V. vinifera* matched the nine orthologous chromosomal regions Itr7, Itr8, Itr14, Itr3, Itr5, Itr12, Itr2, Itr6, and Itr11 in *I. triloba* (Figure 2A and Supplementary Figures 1, 2). These best matched genomic regions showed a median *Ks* of ∼1.20 for anchored gene pairs, which corresponded to the *Ks* peak representing the divergence between *I. triloba* and *V. vinifera* (Figure 1A).

A similar pattern was found in the homologous dotplot between the *S. lycopersicum* and *V. vinifera* genomes. The three paralogous chromosomal regions Vvi1, Vvi14, and Vvi17 generated from ECH in *V. vinifera* matched the nine orthologous chromosomal regions Sly1, Sly4, Sly5, Sly2p, Sly2q, Sly3p, Sly3q, Sly6, and Sly12 in *S. lycopersicum* (Figure 2B and Supplementary Figures 3, 4). These results indicate that the orthologous depth ratios between the *V. vinifera* and *I. triloba*, *V. vinifera*, and *S. lycopersicum* genomes were 3:9 and 3:9, respectively. In a similar manner, it was found that the orthologous depth ratio between the *I. triloba* and *S. lycopersicum* genomes was 3:3 (Figure 2C and Supplementary Figures 5, 6). Combining the genome homologous structure and *Ks* distribution comparisons clearly confirmed that both the *I. triloba* and *S. lycopersicum* genomes experienced one additional whole-genome triplication after the ECH event. These two WGD events in *I. triloba* and *S. lycopersicum* were termed as the SCH and CCH events, respectively (Figure 2D).

*Ks*, the synonymous nucleotide substitutions on synonymous sites, correspond to the amino acid variation. The number of amino acid variants in a genome is positively correlated with its evolutionary rate. Genomes evolved at diverse evolutionary rates (Cui et al., 2006; Wang et al., 2011). The above analyses revealed that the *Ks* peaks of ECH-related paralogs in *V. vinifera*, *S. lycopersicum*, and *I. triloba* were located at 1.054, 1.497, and 1.054, respectively (Figure 1A). This result indicated that the evolutionary rates of the three species were divergent. It was inferred that the *S. lycopersicum* and *I. triloba* genomes evolved 42 and 46% faster than *V. vinifera* genomes after the ECH event, respectively. Significant differences in genome evolutionary rates may lead to distortion when inferring the occurrence times of evolutionary events among considered species. Here, based on an improved version of a previously developed approach (Wang et al., 2015), an evolutionary rate correction was performed by aligning the ECH peaks of three genomes to the same location (Supplementary Table 3) that could decrease the divergence among the evolutionary rates (Wang et al., 2015, 2018a,2019b; Yang et al., 2017). Also, based on the occurrence time of the ECH event ∼115–130 Mya, we found that the SCH event occurred ∼43–49 Mya, the CCH event occurred ∼40–46 Mya, and the divergence of *S. lycopersicum* and *I. triloba* was dated to ∼92–105 Mya (Figure 1B). The calculation of polyploidization event times through evolutionary rate correction could provide more accurate times.

### Event-related multiple genomic alignment framework of the Solanales

**FIGURE 3:**
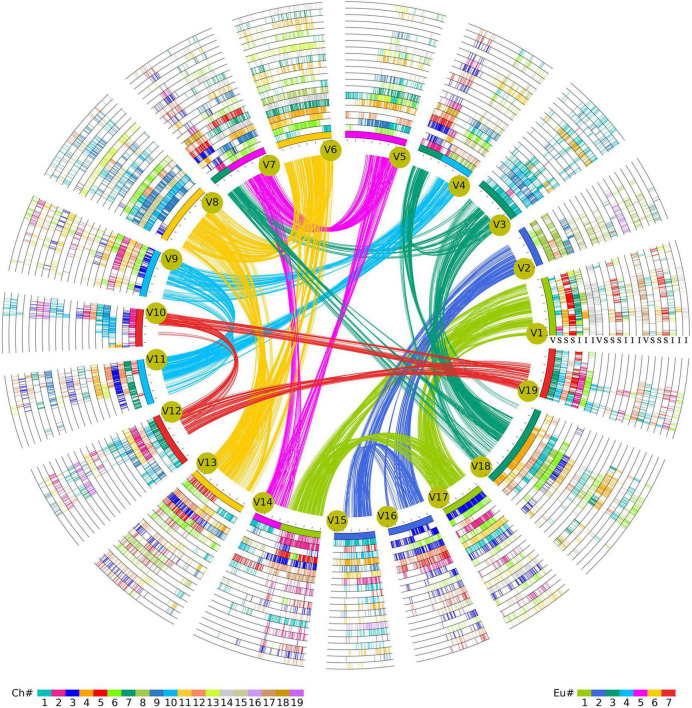
Multigenomic alignment framework for Solanales genomes. Homologous alignments were performed with *Vitis vinifera* (V) as a reference. *V. vinifera* chromosomes form the innermost circle, and their paralogous syntenic genes are linked curves. Exhibiting the information of three plants, including genomic paralogous, orthologous, and outparalogous relationships, each of the three sets of *V. vinifera* paralogous chromosomal regions has three orthologous copies in *Solanum lycopersicum*, forming 3 × 3 = 9 circles, and three orthologous copies in *Ipomoea triloba*, forming 3 × 3 = 9 circles. Finally, 21 circles are formed, each corresponding to Figure 2D. The Ch color scheme at the bottom is based on *V. vinifera*; if the genes of *V. vinifera* and *S. lycopersicum*/*I. triloba* are homologous, lines of corresponding colors are displayed in the circle. Eu corresponds to the colors of the seven chromosomes of the ancestor before the core eudicot common hexaploidization (ECH) event, and the innermost reference genome is the corresponding color of Eu.

According to the information of orthologous and paralogous chromosome regions identified above (Supplementary Tables 4, 5), this study further obtained the event-related homologous genes produced by species divergence and polyploidizations (Supplementary Table 6). It was found that 2,346 paralogous gene pairs involving 3,692 genes were related to ECH events in the *V. vinifera* genome. However, the ECH-related genes within the genomes of *S. lycopersicum* (897 paralogous gene pairs with 1,119 genes) and *I. triloba* (1,200 paralogous gene pairs with 1,782 genes) were less conserved (Supplementary Table 6), which might have been due to the greater number of chromosomal arrangements after the recursive polyploidizations. In addition, statistical analyses of the SCH- and CCH-related homologous genes identified 2,519 paralogous gene pairs involving 4,352 genes related to CCH events in the *I. triloba* genome (Supplementary Table 6). However, analysis identified only 1,505 paralogous gene pairs containing 2,833 genes related to SCH events in the *S. lycopersicum* genome (Supplementary Table 6). These results suggested that *S. lycopersicum* underwent large-scale gene losses after splitting with *I. triloba*. Furthermore, based on the identified genomic homology among the three species, a hierarchical and event-related multiple genome alignment table was constructed using *V. vinifera* as the reference genome (Figure 3 and Supplementary Table 7). The gene IDs of *V. vinifera* were added to the first column, and gene identifiers from *V. vinifera* were added column by column and species by species according to the genome synteny inferred by multiple alignments. Because the SCH event produced three homologies in the *S. lycopersicum* genome, similar to the CCH event in the *I. triloba* genome, each of the three *V. vinifera* paralogous genes derived from the ECH event had three orthologous genes in *S. lycopersicum* and *I. triloba*. Because the syntenic genes that had been lost or translocated did not meet the standards used in the study, a dot was used to fill in the corresponding position. Finally, a 21 = (1 + 3 + 3) × 3 column multiple-genome alignment table was constructed. The Supplementary Table summarizes the results of multiple-genome and event-related alignments, reflecting layers of tripled homology due to recursive polyploidizations. This synteny list is stored in a newly constructed network database created by this research group^1^ and is easily searchable for homologous gene information, providing an essential genomic resource for Solanales.

### Allohexaploid nature of SCH/CCH events and two-step duplication processes

**FIGURE 4:**
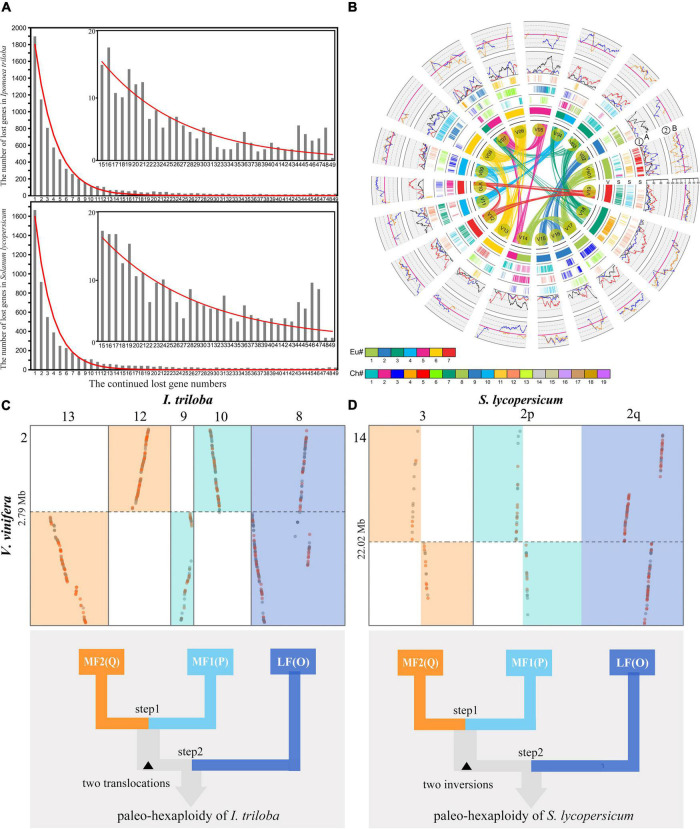
Genomic fractionation and two-step duplications of the Solanaceae common hexaploidization (SCH) and Convolvulaceae common hexaploidization (CCH) events. **(A)** With *Vitis vinifera* as a reference, a geometric distribution was found for the gene loss pattern in *Ipomoea triloba* and *Solanum lycopersicum*. The *x*-axis shows the numbers of continuously missing genes in syntenic regions. The *y*-axis shows the frequency of identified continuously missing gene regions. The subfigure in the upper right shows the fitted geometric distribution of continuously missing genes, with a range of 15–49 genes. **(B)** Genomic alignments and gene retention of *S. lycopersicum* subgenomes along corresponding orthologous *V. vinifera* chromosomes. The genes in 19 chromosomes of *V. vinifera* within the inner circle colored by the seven ancestral chromosomes of core eudicots, as shown in the color scheme at the bottom (denoted by Eu#). Compared to the *V. vinifera* genome, the genomic paralogous and orthologous information within and among the genome of *S. lycopersicum* in subgenomes is displayed in three circles. Each circle is formed by short vertical lines that denote homologous orthologous genes, which are colored to indicate the 12 *S. lycopersicum* chromosome numbers in their respective source plant, as shown in the color scheme at the bottom (denoted by Chr#). ➀ Gene retention level of the least fractionated region (LF, red), the moderately fractionated region (MF1, blue), and the most fractionated region (MF2, grey) in *S. lycopersicum*. ➁ Difference in gene retention between LF and MF1 (pink) and between LF and MF2 (blue). **(C)** Local orthologous blocks identified between the *V. vinifera* and *I. triloba* genomes. Chromosome 2 in the *V. vinifera* genome was generated from one ancestral chromosome of core eudicots after ECH. One chromosome translocation was identified between MF1 and MF2. The bottom Figure expresses the two-step duplication model for the CCH event. **(D)** Local orthologous blocks identified between the *V. vinifera* and *S. lycopersicum* genomes. Chromosome 14 in the *V. vinifera* genome is generated from one ancestral chromosome of core eudicots after the core eudicot common hexaploidization (ECH) event. One chromosome inversion was identified between MF1 and MF2. The bottom Figure shows a two-step duplication model for the SCH event.

Genomic fractionation contributes to reshape genomes, and its main feature is widespread gene loss after recursive polyploidizations (Long et al., 2003; Mitchell-Olds and Schmitt, 2006). To investigate the fractionation patterns of the three subgenomes produced by SCH and CCH events, the gene retention and loss levels of three identified subgenomes in *S. lycopersicum* and *I. triloba* were quantified using *V. vinifera* as a reference. It was found that 55% (774/1,408) and 60% (845/1,408) of genes in *V. vinifera* chromosome 1 were absent from both syntenic locations in the genomes of *S. lycopersicum* and *I. triloba*, respectively (Supplementary Tables 8, 9). In a similar manner, it was found that *S. lycopersicum* and *I. triloba* genomes had the minimum conserved ancestral genes. For example, it was found that only 0.22% (54/24,282) of *S. lycopersicum* SCH-related genes were conserved in all three paralogs, 0.92% (223/24,282) of *I. triloba* CCH-related genes were conserved in all three paralogs, and 2.08% (506/24,282) of *V. vinifera* ECH-related genes were conserved in all three paralogs (Supplementary Tables 10, 11). In addition, this study explored the manner of gene loss in the *S. lycopersicum* and *I. triloba* genomes and found that the lengths and numbers of the continuously removed gene sequences were approximately distributed geometrically (Figure 4A). Approximately half of the runs of genes were 15 or fewer, accounting for 50.2 and 57.2% of all lost genes and up to 24 and 25% of all runs in *S. lycopersicum* and *I. triloba*, respectively. Most of the runs of gene loss were 49 continuous genes or fewer, accounting for 93.1 and 94.9% of all lost genes in *S. lycopersicum* and *I. triloba*, respectively. The distribution of different gene loss regions in *S. lycopersicum* and *I. triloba* was fitted to the geometric distribution curves of different densities, with expansion parameters of 0.3627 and 0.4195, respectively, while *V. vinifera* was used as a reference. The goodness of fit values for *S. lycopersicum* and *I. triloba* were 0.9970 and 0.9955, respectively, and the *P*-values (F test) were 0.9500 and 0.9560, respectively.

To further explore the nature of SCH and CCH events, the genomic fractionation of SCH/CCH-related subgenomes was characterized by counting the levels of gene retention and loss. This study examined the gene retention level between subgenomes related to polyploidization along the 19 chromosomes of the *V. vinifera* genome with the chromosomal sliding window and found that the gene retention showed high differences in almost all local regions on the chromosomes of *S. lycopersicum* and *I. triloba* (Supplementary Figures 7, 8). Furthermore, this study examined the genomic retention levels of each sliding window between any two of three *S. lycopersicum* subgenomes and found that the windows with differences in retention rates >0.05 accounted for more than 70% of all windows (Supplementary Table 12). Similar results were observed in *I. triloba*, and the retention levels of the three subgenomes of *I. triloba* showed significant differences (Supplementary Table 12). This result seemed to suggest that the SCH and CCH events both had an allopolyploid nature. Furthermore, to measure the degree of genomic fractionation between the three subgenomes in *S. lycopersicum* and *I. triloba*, this study employed the previously developed P-index statistical indicator (Wang et al., 2019a). According to previous studies, autopolyploidies and allopolyploidies can be distinguished due to the P-index threshold of 0.3; for example, *B. napus*, *Z. mays*, *G. hirsutum*, and *B. oleracea* were inferred to have allopolyploidy, while the P-index was >0.3 (Schnable et al., 2011; Chalhoub et al., 2014; Barker et al., 2016; Wang et al., 2018b). It was estimated that the P-index values of the *S. lycopersicum* and *I. triloba* hexaploid ancestor were 0.81 and 0.53, respectively (Supplementary Table 12). Therefore, it was inferred that the genomes of *S. lycopersicum* and *I. triloba* were all allopolyploid and originated from SCH and CCH events, respectively.

As allopolyploids, the genomes of *S. lycopersicum* and *I. triloba* showed obvious genomic fractionation bias. Here, according to the syntenic relationship between *V. vinifera* and *S. lycopersicum* and *I. triloba*, the three orthologous regions in the *S. lycopersicum* and *I. triloba* genomes were identified. Comparing the gene retention level across each triplicated region demonstrated that all 21 regions showed significant imbalance (Supplementary Figures 9, 10). For example, the three orthologous regions of chromosome 3 in *V. vinifera* were located on chromosomes 1, 2, and 10 in *S. lycopersicum*. The numbers of retained genes on chromosomes 1, 2, and 10 in *S. lycopersicum* were 356, 18, and 96, respectively (Supplementary Table 9), indicating a high degree of divergence and obvious genomic fractionation bias. The gene retention difference between the corresponding paralogous regions of the dominant and two sensitive subgenomes always varied away from zero (Figure 4B). A similar result was observed in the *I. triloba* genome (Supplementary Figures 7, 8). We defined the three subgenomes as the least fractionated region (LF), the moderately fractionated region (MF1), and the most fractionated region (MF2). Therefore, these comparisons suggested that the SCH and CCH events were generated from two-step duplications.

The duplication process of allopolyploidy was further explored using the genomic structural comparisons. A large chromosomal translocation was identified that probably occurred in the process of two-step duplications in the *I. triloba* genome. Based on the genomic homologous relationship between *V. vinifera* and *I. triloba*, one integrated orthologous region of chromosome 2 (Vvi2) in *V. vinifera* was found to be located in chromosome 8 (Sly8) of *I. triloba*, whereas the other orthologous regions were located in the separate chromosomes of Itr9, Itr10, Itr12, and Itr13 (Figure 4C and Supplementary Figure 1). Interestingly, the regions located in Itr9 and Itr10 and the regions located in Itr12 and Itr13 were complementary chromosome breakages that were caused by chromosomal translocations during the evolution of *I. triloba*. The breakpoints of Itr9 and Itr10 were shared with Itr12 and Itr13, and the breakpoint located in chromosome Vvi2 was found at ∼2.72 Mb, suggesting that this translocation possibly occurred in the ancestral chromosome of these two pairs of complementary chromosome regions. Additionally, it was found that these two pairs of complementary chromosome regions were located in the identified subgenomes of MF1 and MF2 (Figure 4C). Using similar methods, one chromosomal inversion that occurred in the *S. lycopersicum* genome was identified. For Vvi14 in *V. vinifera*, the three orthologous regions were located in Sly2p, Sly2q, and Sly3. The lower half of Sly2p and Sly3 was reversed 180° compared to the upper half, whereas this pattern did not appear in another orthologous region of Vvi14 located in Sly2q in *S. lycopersicum* (Figure 4D and Supplementary Figure 2). The breakpoint of Sly2p was shared with that of Sly3, and this study identified the breakpoints located on chromosome Vvi2 at ∼22.02 Mb, suggesting that this inversion occurred in the ancestral chromosomes of these two pairs of complementary chromosome regions. Similar to that in *I. triloba*, structural variation also occurred in the identified subgenomes MF1 and MF2 (Figure 4D). These genomic rearrangement comparisons suggest that the subgenomes MF1 and MF2 may share a closer common ancestor than LF and provide evidence that the ancient hexaploidizations of Solanaceae and Convolvulaceae were generated from two-step duplications. More importantly, it was concluded that the subgenomes of MF1 and MF2 occurred in a tetraploid as the first step in two-step duplications of SCH and CCH and then hybridized with the subgenome of LF1 as the second step.

### Evolution of the BMY gene family

**FIGURE 5:**
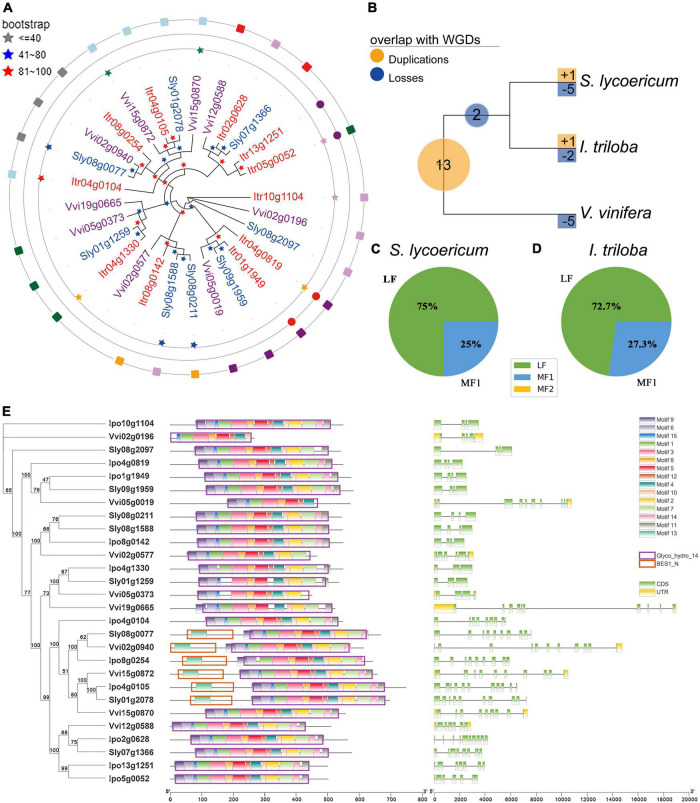
Evolution and structural diversification of β-amylase (BMY) genes in the studied genomes. **(A)** Phylogenetic tree of BMY genes. The bootstrap values that indicate the credibility levels of the structures are shown by the different color stars. The genes with different colors belong to different species, the three circles are located represent the different relationships between genes (inner to outer: paralogous, paralogous, and orthologous respectively), the same color or the same shape in the same circle represents the same kind of relationship. **(B)** Gene duplications and losses at different periods along the evolutionary history of studied genomes. **(C)** Distribution of BMY genes in each of the three subgenomes [least fractionated region (LF), moderately fractionated region (MF1), and most fractionated region (MF2)] within *Solanum lycopersicum*. **(D)** Distribution of BMY genes in each of the three subgenomes (LF, MF1, and MF2) within *Ipomoea triloba*. **(E)** Structural [motif, domain, coding sequence (CDS), and untranslated region (UTR)] distribution of BMY family members. The unit used for motifs 1–15 and domains (Glyco_hydro_14 and BES1_N) was amino acid, and the unit used for CDSs and UTRs was bp. WGD, whole-genome duplication.

Amylum is one of the main components of *I. triloba*, and BMY genes contribute to amylum accumulation and storage root swelling in *I. triloba*. Nine, eight, and eleven BMY genes were identified in the genomes of *V. vinifera*, *S. lycopersicum*, and *I. triloba*, respectively (Figure 5A). Furthermore, the homologous relationships were obtained between the BMY genes in the three species. Then, combined with the identified homology information and the phylogenetic relationship of genes in families, we calculated the whole-genome duplication expansion rate (*WGD-ER*), tandem duplication expansion rate (*TD-ER*), and the contraction rate (*CR*) caused by gene loss for each family. For *S. lycopersicum*, it has a duplication expansion rate 75% (*WGD-ER*: 75% and *TD-ER*: 0%, respectively), which is lower than that the *CR* with 200% (Figure 5A). For *I. triloba*, it has a duplication expansion rate 100% (*WGD-ER*: 100% and *TD-ER*: 0%, respectively), which is also lower than that the *CR* with 175%. We also found that the BMY in *V. vinifera* has *TD-ER* to be 12.5% (Figure 5A). In addition, the processes of the duplications and losses of the BMY gene family were inferred through phylogenetic analysis using NOTUNG software, and a total of 13 BMY gene duplications were found in the ancestral genome of *V. vinifera*, *S. lycopersicum*, and *I. triloba* after the ECH event (Figure 5B). Notably, many BMY genes (five genes) were lost in *V. vinifera*, which might also imply that there were many BMY genes lost in *S. lycopersicum* and *I. triloba* (Figure 5B).

To further investigate the effects of the SCH/CCH events on the evolution of the BMY gene family, the BMY genes were located in the subgenomes (LF, MF1, and MF2) of *S. lycopersicum* and *I. triloba* (Supplementary Table 13). Most BMY genes were found in the subgenome LF (Figures 5C,D). For example, 75% (6/8) BMY genes belonged to LF, 25% (2/8) of BMY genes belonged to MF1, and none of the BMY genes belonged to MF2 in the *S. lycopersicum* genome (Figure 5C). 72.7% (8/11) BMY genes belonged to LF, 27.3% (3/11) of BMY genes belonged to MF1, and none of the BMY genes belonged to MF2 in the *I. triloba* genome (Figure 5D). These analyses revealed that the second step duplication of SCH/CCH made an important contribution to the expansion of the BMY family. Furthermore, to compare the genetic diversity of important trait genes in Solanaceae and Convolvulaceae, the domain, motif, and gene structures of BMY genes among the three species were analyzed. The Glyco_hydro_14 domains of BMY family proteins contained different conserved motifs, with most containing motifs 1–12 and 14 (Figure 5E and Supplementary Table 13). The BMY genes in *S. lycopersicum* had more structural diversity than those in *I. triloba*; for example, in *S. lycopersicum*, 50% (4/8) of BMY genes had structural changes, while only 27% (3/11) had structural changes in *I. triloba*. Based on the CDS analysis of BMY genes, it was found that most of the genes which contains more than four CDSs were derived from the LF subgenomes (Figure 5E). For example, all *I. triloba* genes containing more than four CDSs were derived from LF (100%, 6/6), and 66.67% (2/3) of the genes in *S. lycopersicum* were derived from the LF (Figure 5E). Therefore, it was inferred that the joining of the LF subgenome in the two-step duplications of SCH/CCH possibly promoted the diversity of the family gene structures.

Furthermore, in order to explore whether the different structures resulted in the functional differences, this study analyzed the subcellular localization of BMY proteins in selected genomes. Compared with *I. triloba*, the localization of BMY proteins in *S. lycopersicum* was more diverse (Supplementary Table 13). For example, there were two types of subcellular localizations (extracell and extracell/nucleus) for BMY proteins in *I. triloba* but four types (extracell, extracell/nucleus, lysosome, and cytoplasm/extracell) in *S. lycopersicum* (Supplementary Table 13). Comparing to the BMY genes in MF1 genomes of *S. lycopersicum* and *I. triloba*, there were one extra types (extracell/nucleus) only occurred in *I. triloba* LF subgenomes, and two (lysosome and cytoplasm/extracell) in *S. lycopersicum* (Supplementary Table 13).

## Discussion

### Two independent polyploidizations in Solanales

Recursive polyploidization occurs frequently in plants and has been considered a tremendous force in angiosperm diversification (Jiao et al., 2011; Song et al., 2020; Wu et al., 2020). Chromosomal rearrangements after polyploidization increase the complexity of the genome and make it difficult to decipher their polyploidization events (Zhuang et al., 2019). In a study of *S. tuberosum*, the author reported that Solanaceae experienced a tetraploidization event before ∼67 Mya (Xu et al., 2011). However, the studies on the genomes of *Capsicum annuum* (Barchi et al., 2019), *Lycium chinense* (Cao et al., 2021), *Solanum melongena* (Barchi et al., 2019), and *S. tuberosum* (Xu et al., 2011) suggested that the polyploidization events of these genomes were hexaploidization events that occurred at ∼89 Mya (Xu et al., 2011), ∼69 Mya (Cao et al., 2021), and ∼67 Mya (Xu et al., 2011), respectively. Furthermore, it was inferred that the large number of chromosomal blocks lost led to the serious genome fractionation. This results in the 1:3 of syntenic depth ratio between the reference genome (*V. vinifera*) and *S. tuberosum* genome, which was not obvious in the study of *S. tuberosum* (Xu et al., 2011) and misled the authors to infer that the recent polyploidization of *S. tuberosum* was a tetraploidization event.

To clarify the polyploidization histories in Solanales, using the pipeline previously proposed (Yang et al., 2017; Wang et al., 2018a,^2020^), we re-analyzed the genomes of *S. lycopersicum* and *I. triloba*. We found that the syntenic depth ratios were 1:3, 1:3, and 3:3 of *V. vinifera–S. lycopersicum*, *V. vinifera–I. triloba*, and *S. lycopersicum–I. triloba*, respectively, strongly indicating that independent polyploidizations of Solanaceae and Convolvulaceae occurred after ECH. Furthermore, it was found that the hexaploidization events of two families in Solanales occurred at ∼43–49 and ∼40–46 Mya, respectively, which was consistent with previous research results on *S. melongena* and *I. batatas* (Wu et al., 2018). In addition, our result showed that the genomes *S. lycopersicum* and *I. triloba* evolved more rapidly than *V. vinifera* genome. The significant differences in evolutionary rates of plant genomes may lead to distortion when inferring the occurrence times of evolutionary events. The previous studies may not have accurately estimated the times of polyploidization events, and the *S. lycopersicum* and *I. triloba* are not the only plants in which the evolutionary histories of polyploidization events have been misinterpreted. The other misinterpretations have also been reported in studies on the genomes of cotton, durian, and carrot (Meng et al., 2020; Wang et al., 2019b,^2020^). In this study, we clarified the evolutionary history of Solanales, and analyzed the differences of evolutionary rates among studied species, providing a solid genomic basis for further understanding the evolutionary history of Solanales following the recursive polyploidization events.

### An excellent multigenomic alignment framework for Solanales

Complex genomic structures of plants make it difficult to decipher their genome homology, understand their genome evolutionary trajectories, and explore the evolution of genes or regulatory pathways of important traits. The number of event-related genes may help to reflect the effects of different events on genome expansion and differentiation (Soltis et al., 2015). Based on this possibility, a multiple genome alignment table was constructed that contained the *S. lycopersicum* and *I. triloba* genomes with *V. vinifera* as an appropriate reference genome. This effort was valuable in deconvoluting the layers of homologous regions packed together after recursive polyploidizations, producing a list of homologous genes, paralogs, and orthologs and relating these homologs to each ancestral polyploidization event. The list represents how and when a pair of homologs were produced and diverged and whether gene deletion occurred after certain events, providing valuable data to help reveal the evolutionary and functionally innovative trajectories of genes, gene families, regulatory pathways, and economically and agriculturally important traits. For botanist studying the other species of Solanales, they can add the homologous genes into the current multigenomic alignment framework to expand the application of homologous gene list. Furthermore, we have built database resources (see text footnote 1) which can provide clear gene homology information for researchers who are not specialized in bioinformatics. For example, using the “synthetic list” function of built-in database, researchers can search the genes with a specific function of interest to understand their origin and evolution. This multiple genome alignment table provides an excellent multigenomic alignment framework for the future study of Solanales.

### Genomic fractionation and two-step duplications of SCH/CCH

Recurrent polyploidization plays a key role in the evolution of the large plant kingdom and contributes to artificial selection during crop domestication (Jiao et al., 2011; Cheng et al., 2018; Wang et al., 2019a; Meng et al., 2020; Cai et al., 2021). Allopolyploidization and autopolyploidization are two types of polyploidization. Allopolyploids are formed by hybridization between different genomes, while autopolyploidies are formed by genomic duplication events (Doyle et al., 2008; McCarthy et al., 2019). Notably, allopolyploid events may promote heterosis, which makes plants more adaptable to their environment and allows them to form large populations (Wang et al., 2019a). In this study, we found that the biased fractionation between polyploidizations produced subgenomes in *S. lycopersicum* and *I. triloba* (Figure 4A and Supplementary Tables 8–11), respectively, suggesting that the allopolyploidy nature of the hexaploidization events in Solanaceae and Convolvulaceae. Allopolyploidizations may have increased the evolutionary rates of species due to homologous recombination and the presence of additional gene and chromosome copies, as demonstrated in studies on wheat, maize, soybean, cotton, tobacco, strawberry, and oilseed rape (Schnable et al., 2011; Chalhoub et al., 2014; Barker et al., 2016; Wang et al., 2018b). Additionally, the allopolyploidization events may promote to the formation of large plant families (Wang et al., 2019a). Therefore, we inferred that the two independent allohexaploidizations were responsible for the establishment of two largest families Solanaceae and Convolvulaceae in Solanales. In particular, we found that the biased genomic fractionation of *S. lycopersicum* was slightly stronger than that of *I. triloba*, which seems to explain why Solanaceae has more species diversity than Convolvulaceae.

Hexaploidization events are very rare compared with ordinary duplication. As a classic hexaploidization event, the ECH event covered 75% of angiosperms (Ren et al., 2018; Zeng et al., 2014). Although the formation process remains uncertain, the findings of the present study have confirmed the two hexaploidization events in Solanales. In previous studies, researchers have proposed that the formation process of hexaploidy is best explained by a two-step fractionation model, such as the ECH event (Jaillon et al., 2007), *Brassica* common hexaploidy (Wang et al., 2011; Tang et al., 2012). One potential reason for this observed gene retention bias among these genomes is the fact that two of the genomes were in the same nucleus for a significantly longer period than the third. Alternatively, one of the three genomes was naturally more resistant to fractionation than the other two. These possibilities do not necessarily apply to all hexaploid species (Lyons et al., 2008). The analysis of genomic structural variations may help to clarify the evolutionary history of polyploidization events because rare genomic changes have more alternative states and may be less vulnerable to the high frequency of reversals or parallel substitutions in sequence evolution (Qin et al., 2021). Notably, through genomic fractionation and structural variation comparisons, the duplication orders of SCH/CCH-produced subgenomes were inferred for two paleohexaploidizations, and the previous hypothesis that the most recently added set of subgenomes would be dominant was supported (Lyons et al., 2008; Wang et al., 2011). Genomic structural variation comparisons may be the beginning of a new era in understanding evolutionary events that occurred in a very compressed evolutionary window, such as the ancient allohexaploidization event.

### Genetic diversity of important trait genes

The understanding of the roles of β-amylase (BAM) gene family members in plants has increased dramatically with the continuous development of genomics. BMY occupies a significant position in the catalog of enzymes of industrial importance owing to its saccharogenic activities, which are of immense importance in the pharmaceutical and food industries (Ray and Nanda, 1996; Raveendran et al., 2018). BMY genes break starch into maltose during fruit ripening, resulting in a sweet flavor (Streb and Zeeman, 2012; Ampa et al., 2017). Based on the significant function of BMY genes in plants, BMY genes have been analyzed in many genomes, such as those of *A. chinensis* (Ampa et al., 2017), *Arabidopsis thaliana* (Lao et al., 1999; Monroe and Storm, 2018; Nag et al., 2021), *G. max* (Hirata et al., 2004), and *I. batatas* (Zhu et al., 2021). In the present study, we explored the expansion patterns of BMY genes. Our findings revealed that the WGD events provided the genetic basis for the expansion of the BMY family in Solanales, but the gene loss may be inhibiting the family expansion. We also found that the second-step duplication of SCH/CCH may be contributed to the expansion of BMY genes.

Variations in structures may result in functional differences. It is known that genes, as the units of genetic information, constitute the basis of the mRNA and protein products detected in living organisms (Finta et al., 2002), and changes in gene structure may cause offspring to exhibit new traits, thereby promoting the emergence of a new species or strengthening the survival adaptability of a species. In our study, the genomic fractionation within *S. lycopersicum* is slightly stronger than that of *I. triloba*, and the differences among the motifs or CDS structure of *S. lycopersicum* genes are stronger than those of *I. triloba* (Figure 5E). We also found that the structural diversity of BMY genes were strongly influenced by the second-step duplication of SCH/CCH event in *S. lycopersicum* and *I. triloba*. Although the allopolyploidization events limit the family expansion, we suggested that it like a booster for the structural diversity of family genes. The reason for this speculation is that the biased subgenome fractionation of species may require more structural variations of genes to compensate for their adaptability to the environment. Besides, the subcellular localization showed that the differences among structures (CDS, UTR, domain, and motif) resulted in diversity of functional sites of the BMY genes. Our findings provide new clues to trace the evolution of Solanales BMY gene family, and could facilitate the further investigate the breeding of Solanales BMY genes in the crops saccharogenic activities.

## Materials and methods

### Materials

The genomes and annotation files for each genomic project were downloaded from different websites. The genomic data of *V. vinifera* were obtained from the JGI database,^2^ and the *S. lycopersicum* genome assembly and annotations were downloaded from the Genome Sequence Archive (GSA) database^3^ in the BIG Data Center (accession number PRJCA004585). The *I. triloba* genomic data were obtained from the GenBank database^4^ (accession number PRJNA428241).

### Inferring genomic synteny

To identify the duplicated genes produced by SCH and CCH and the orthologous genes related to the speciation of the considered genomes, the potential homologous gene pairs were searched using BLASTP (Altschul, 2012), with the strict parameters of an E-value < 1e-5 and score > 100. Then, gene homology information was used as an input to ColinearScan (Wang et al., 2006) to infer the syntenic gene pairs and examine the significance of the synteny of chromosomal regions (blocks), while the key parameter, the maximum gap, was set to 50 intervening genes. The large gene families with 50 or more members were removed from the blocks. Finally, genomic homologous structural analyses were performed through homologous dotplots to help determine the paralogous and orthologous genes. This genome synteny analysis approach has been adopted in many previous angiosperm genomic comparisons (Yang et al., 2017; Wang et al., 2018a,2019b).

### Calculation of *Ks*

First, multiple gene coding sequences (CDSs) were translated to proteins, and the proteins were aligned using ClustalW with default parameters (Thompson et al., 2002). The proteins were then translated to DNA, which was used to calculate the synonymous nucleotide substitutions on synonymous sites (*Ks*) between homologous gene pairs. *Ks* values were estimated using the Nei–Gojobori approach (Nei and Gojobori, 1986) implemented with the Bioperl Statistical module.

The *Ks* distributions of homologous genes between and within different genomes can infer the timings of species divergence and important polyploidization events. The kernel function was used to analyze the *Ks* distribution of syntenic homologs within and between genomes. The *Ks* distribution is considered a mix of normal distributions (Cichosz et al., 2020). MATLAB was used to estimate the density of each *Ks* list and obtain the density distribution curves, while the width parameter of the kernel smoothing density function *Ks* density was set at 0.05. The curve was fitted by the Gaussian method in the fitting toolbox cftool. The R-squared parameter used to evaluate the goodness of fit was generally set to at least 95%, the smallest number of normal distributions was used to represent the complex *Ks* distribution, and the corresponding evolutionary event was represented by one peak. The maximum likelihood estimate μ (*Ks* peak) from the *Ks* distribution curves was used.

### Correction of evolutionary rates

Based on the previously developed approach (Wang et al., 2015, 2018a,2019b; Yang et al., 2017), we updated and established an algorithm to adapt to the evolutionary rate correction in this study. To correct the evolutionary rates of ECH-produced duplicated genes, the maximum likelihood estimate μ from the inferred *Ks* means of ECH-produced duplicated genes was aligned to have the same value as that of *V. vinifera*, which underwent the slowest evolution. Supposing that a *V. vinifera* duplicated gene pair had a *Ks* value that was a random variable ${\text{X}}_{\text{G}}:\left(\mu_{\text{G}},\sigma_{\text{G}}^{2}\right)$ and a duplicated gene pair in another genome had a *Ks* of ${\text{X}}_{\text{i}}:\left(\mu_{\text{i}},\sigma_{\text{i}}^{2}\right)$, the relative difference was


$$\text{r}=(\mu_{\text{i}}-\mu_{\text{G})}/\mu_{\text{G}}$$

Then, rate correction was performed to find the corrected rate ${\text{K}}_{\text{i}-\text{correction}}:\left(\mu_{\text{i}-\text{correction}},\sigma_{\text{i}-\text{correction}}^{2}\right)$ of the other evolutionary node *i* related to μ_G_.

(1) For *S. lycopersicum*, using the *Ks* between its ECH duplicates, a correction coefficient λ_i_ can be defined as


$$\frac{\mu_{\text{i}-\text{correction}}}{\mu_{\text{i}}}=\frac{\mu_{\text{G}}}{\mu_{\text{i}}}=\lambda_{\text{i}}$$

Therefore, the following is obtained:


$$\mu_{\text{i}-\text{correction}}=\frac{\mu_{\text{G}}}{\mu_{\text{i}}}\times \mu_{\text{i}}=\frac{1}{1+\text{r}}\times \mu_{\text{i}},$$

$$\lambda_{\text{i}}=\frac{1}{1+\text{r}}$$

(2) For *I. triloba s*, the *Ks* between its ECH duplicates was similar to that of *S. lycopersicum z*, and the correction coefficient λ_s_ could be defined as


$$\lambda_{\text{s}}=\frac{1}{1+\text{r}}$$

(3) For *Ks* between homologous genes between *V. vinifera y* and *S. lycopersicum z*, it was supposed that the *Ks* distribution was ${\text{X}}_{\text{yz}}:\left(\mu_{\text{yz}},\sigma_{\text{yz}}^{2}\right)$. The study then adopted the algebraic mean of the correction coefficients from the two plants:


$$\lambda_{\text{yz}}=\frac{\lambda_{\text{y}}+\lambda_{\text{z}}}{2}$$

If the peak was located at K_yz_, supposing the correction coefficient λ_yz_ between *V. vinifera y* and *S. lycopersicum z*, a corrected evolutionary rate was calculated as


$$\mu_{\text{yz}-\text{correction}}=\lambda_{\text{yz}}\times \mu_{\text{yz}}$$

(4) For *Ks* between homologous genes from *V. vinifera y* and *I. triloba*, it was supposed that the *Ks* distribution was ${\text{X}}_{\text{ys}}:\left(\mu_{\text{ys}},\sigma_{\text{ys}}^{2}\right)$. The study then adopted the algebraic mean of the correction coefficients from the two plants:


$$\lambda_{\text{ys}}=\frac{\lambda_{\text{y}}+\lambda_{\text{s}}}{2}$$

If the peak was located at μ_ys_, supposing the correction coefficient λ_ys_ between *V. vinifera y* and *I. triloba*, a corrected evolutionary rate was calculated as


$$\mu_{\text{ys}-\text{correction}}=\lambda_{\text{ys}}\times \mu_{\text{ys}}$$

(5) For *Ks* between homologous genes from *S. lycopersicum z* and *I. triloba*, it was supposed that the *Ks* distribution was ${\text{X}}_{\text{zs}}:\left(\mu_{\text{zs}},\sigma_{\text{zs}}^{2}\right)$. The study then adopted the algebraic mean of the correction coefficients from the two plants as follows:


$$\lambda_{\text{zs}}=\frac{\lambda_{\text{z}}+\lambda_{\text{s}}}{2}$$

If the peak was located at μ_zs_, supposing the correction coefficient λ_zs_ between *S. lycopersicum z* and *I. triloba*, a corrected evolutionary rate was calculated as


$$\mu_{\text{zs}-\text{correction}}=\lambda_{\text{zs}}\times \mu_{\text{zs}}$$

(6) For *Ks* within *S. lycopersicum z* homologous genes related to the SCH event, it was supposed that the *Ks* distribution was ${\text{X}}_{\text{z}-\text{SCH}}:\left(\mu_{\text{z}-\text{SCH}},\sigma_{\text{z}-\text{SCH}}^{2}\right)$. The study then adopted the algebraic mean of the correction coefficients from the two plants:


$$\lambda_{\text{z}-\text{SCH}}=\frac{1}{1+\text{r}}$$

If the peak was located at μ_z−SCH_, supposing the correction coefficient λ_z−SCH_ within *S. lycopersicum z*, a corrected evolutionary rate was calculated as


$$\mu_{\text{z}-\text{SCH}-\text{correction}}=\mu_{\text{z}-\text{SCH}-\text{correction}}\times \frac{\mu_{\text{z}-\text{ECH}}}{\mu_{\text{z}-\text{SCH}}}$$

(7) For *Ks* within *I. triloba* homologous genes related to the CCH event, it was supposed that the *Ks* distribution was ${\text{X}}_{\text{s}-\text{CCH}}:\left(\mu_{\text{s}-\text{CCH}},\sigma_{\text{s}-\text{CCH}}^{2}\right)$. The study then adopted the algebraic mean of the correction coefficients from the two plants:


$$\lambda_{\text{s}-\text{CCH}}=\frac{1}{1+\text{r}}$$

If the peak was located at μ_s−CCH_, supposing the correction coefficient λ_s−CCH_ within *I. triloba s*, the corrected evolutionary rate was calculated as


$$\mu_{\text{s}-\text{CCH}-\text{correction}}=\mu_{\text{S}-\text{CCH}-\text{correction}}\times \frac{\mu_{\text{s}-\text{ECH}}}{\mu_{\text{s}-\text{CCH}}}$$

### Construction of the event-related synteny gene table

A multiple-genome alignment table was constructed using the *V. vinifera* genome as a reference. When the first column was filled with the *V. vinifera* genes, each *V. vinifera* gene had two extra syntenic genes due to the ECH event. Two additional columns in the table were used to contain this information. For each *V. vinifera* gene, when a corresponding syntenic gene appeared in an expected location, a gene ID was filled in a cell of the corresponding column in the table. When it was missing, often due to gene loss or translocation in the genome, a dot was added to the cell. For the *S. lycopersicum* genome, there were a total of 3 × 3 columns due to extra hexaploidy. *I. triloba* also has extra hexaploidy, similar to that of *V. vinifera*, and has a nine-column gene ID. Therefore, the obtained multiple-genome alignment table had 21 columns reflecting ploidy changes caused by different polyploidization events among the three species.

### Calculation of the polyploid index (p-index)

To infer the possible nature of polyploidy, the degree of differentiation between polyploid subgroups was evaluated using the previously developed statistical data corresponding to the P-index (Wang et al., 2019a). Previous studies demonstrated the robustness of P-indices for “diagnosing” the nature of polyploidies, while plants with P-indices > 0.3, such as *Brassica napus*, *Zea mays*, *Gossypium hirsutum*, and *Brassica oleracea*, were inferred to be paleoallopolyploids (Schnable et al., 2011; Wang et al., 2018a,2019b; Cai et al., 2021). In contrast, the genomes of *Glycine max*, *Populus trichocarpa*, and *Actinidia chinensis* were inferred to be paleoautopolyploid due to a P-index < 0.3 (Yang et al., 2017; Wang et al., 2019a). The detailed P-index calculation scheme is described below.

This study used *V. vinifera* as a reference and calculated the P-index among the three subgenomes of the *S. lycopersicum* and *I. triloba* genomes, identifying the potential gene losses or translocations in each of the inferred subgenomes produced by an ancient polyploidization event. The P-index was calculated between the subgenomes of *S. lycopersicum* and *I. triloba* with the number of sliding windows set to 50 and a parameter of 0.1–1. Based on a P-index demarcation line of 0.3 that distinguished between known or previously inferred allo- or autopolyploidies, assuming that there were K chromosomes in the reference genome, subgenomes A and B were identified in the genome of interest. Regardless of whether one dominated, each pair of homoeologous chromosomes was divided into N_c_ windows with M (such as 10) genes. For the i-th window of a specific homoeologous chromosome pair, the gene retention rates Ai and Bi relative to the reference genome were obtained; thus, the P-index value was determined as follows:


$$\text{P}-\text{index}=\sum_{\text{c}=1}{\text{W}}_{\text{c}}\,\text{abs}\,\left[\frac{\sum_{\text{i}=1}^{{\text{N}}_{\text{c}}}\frac{{\text{A}}_{\text{i}}-{\text{B}}_{\text{i}}}{\text{abs}\,\left({\text{A}}_{\text{i}}-{\text{B}}_{\text{i}}\right)}\times \delta_{\text{i}}}{{\text{N}}_{\text{c}}-\delta }\right]$$

More details are provided in a previous article (Wang et al., 2019a).

### Identification and evolutionary analyses of BMY genes

To identify the BMY family proteins involved in amylum accumulation and storage root swelling, the Glyco_hydro_14 domain (PF01373) was downloaded from the PFAM database hidden (Mistry et al., 2021), and the Markov model (HMM) program of HMMER v3.3 (Potter et al., 2018) was employed to search the BMY proteins from the genomes of *V. vinifera*, *S. lycopersicum*, and *I. triloba*, and setting a strict expectation (E-value < 1e–10). For phylogenetic analysis of the BMY family, we performed a multiple sequence alignment on the BMY proteins using MAFFT software (Standley, 2013), and constructed the gene tree using the IQ-Tree software (Lam-Tung et al., 2015). The algorithm used was the maximum likelihood (ML) method with 1,000 bootstraps. The model of each family was JTTDCMut+G4, which was the best model for IQ-tree matching (MFP). The results were visualized by the Evolview website (Subramanian et al., 2019). Moreover, for the expansion rates of the whole-genome and tandem duplication (*WGD-ER* and *TD-ER*) and contraction rates (*CR*) of the family in Solanales, supposing that the family of *V. vinifera* has *N* = [(*total number of genes*) – (*number of the newly gained genes from tandem duplication*)] genes, we designed the following algorithm:

(1) *WGD-ER* = (*total number of the newly gained genes from polyploidizations* / *N*) × 100%
(2) *TD-ER* = (*number of the newly gained genes from tandem duplication* / *N*) × 100%
(3) *CR* = (*number of V. vinifera genes lost in Solanales* / *N*) × 100%

Furthermore, to explore the evolutionary history of family members, NOTUNG (Chen et al., 2000) software was used to predict gene duplications and losses at phylogenetic nodes in the species (*V. vinifera*, *S. lycopersicum*, and *I. triloba*) tree and the phylogenetic tree of family members. The results were illustrated using the Evolview website.^5^

Next, to explore the structural diversification of BMY genes, the conserved motifs of BMY family proteins were detected by the MEME database (Bailey and Elkan, 1994), and the domains were detected by the PFAM database (Mistry et al., 2021). The CDSs and untranslated regions (UTRs) of the family genes were analyzed using the online tool GSDS (Hu et al., 2015). In addition, the subcellular localization of BMY proteins, including those with multiple sites, was predicted by the Euk-mPLoc 2.0 database (Chou and Shen, 2010).

## Data availability statement

The datasets presented in this study can be found in online repositories. The names of the repository/repositories and accession number(s) can be found in the article/Supplementary material.

## Author contributions

JpW and LW conceived and led the research. YZ implemented and coordinated the analysis. JZ, QXu, ZY, SB, JyW, and YL performed the analysis. JpW, LW, YZ, LZ, QXi, and CW wrote the manuscript. All authors contributed to the article and approved the submitted version.

## Footnotes

1
http://www.cgrpoee.top/Solanales/index.html

2
https://phytozome-next.jgi.doe.gov/info/Vvinifera_v2_1

3
http://bigd.big.ac.cn/gsa

4
https://www.ncbi.nlm.nih.gov/

5
https://www.evolgenius.info/evolview/#/
