# Supplementary material for: Two independent allohexaploidizations and genomic fractionation in Solanales
Source: Front Plant Sci. 2022 Sep 23;13:1001402. doi: 10.3389/fpls.2022.1001402 (PMC9538396; doi:10.3389/fpls.2022.1001402)
Supplement: Supplementary file 1 [file Data_Sheet_1.docx]

**Supplementary figures 1-11
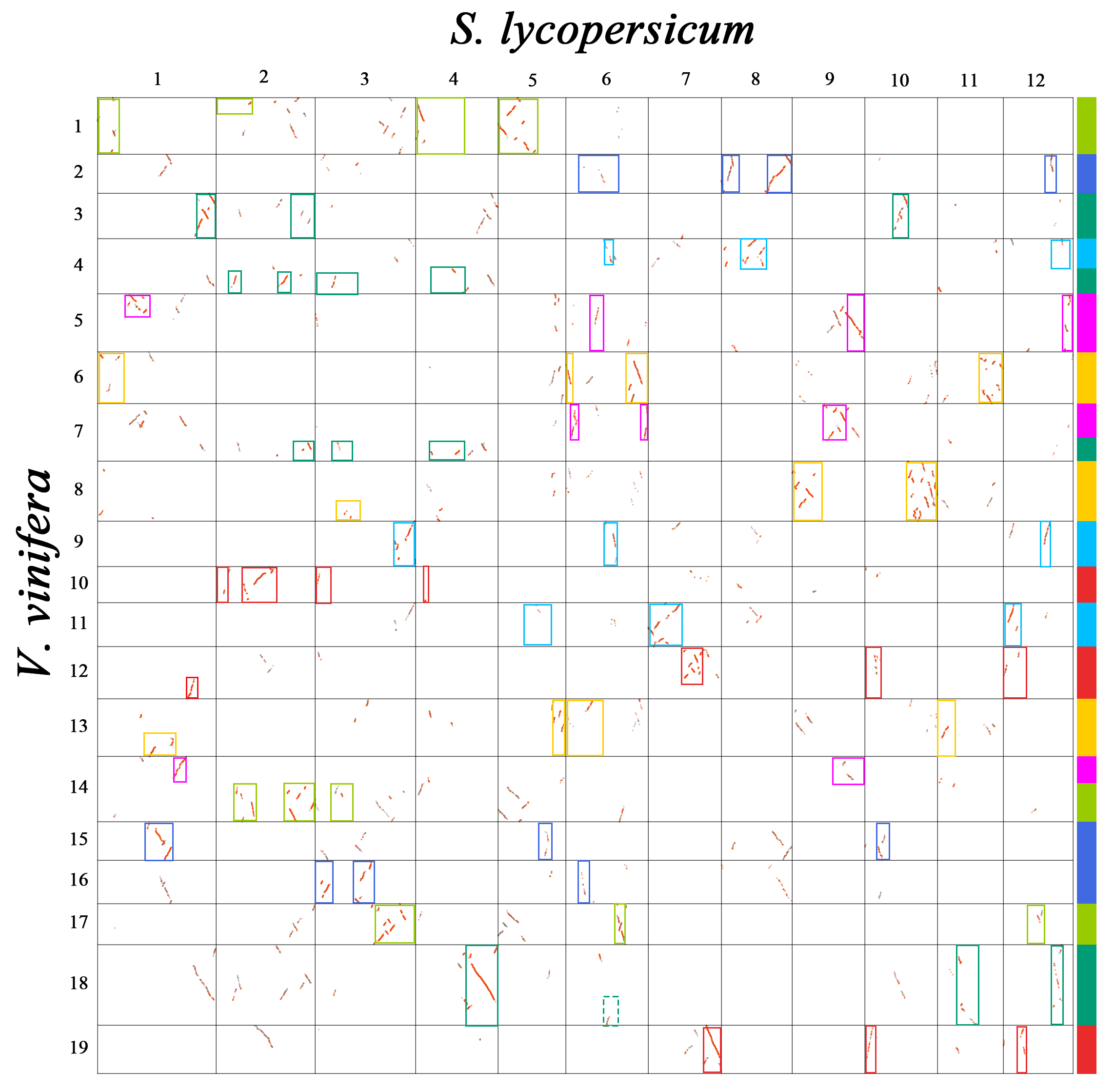
**

**Fig S1. Intergenomic homologous structure between the genome of *Vitis vinifera* and *Solanum lycopersicum*.**

The best, secondary, and other matched homologous gene pairs output by Blastp were plotted by red, gray, and gray colors in this figure, respectively. The dotplot shows only relatively large orthologous regions (containing more than 10 gene pairs). The highlighted box next to the *V. vinifera* chromosome corresponds to the seven colors of the eudicot’s ancestor, and the part with highlighted color in solid line frame is orthologous.


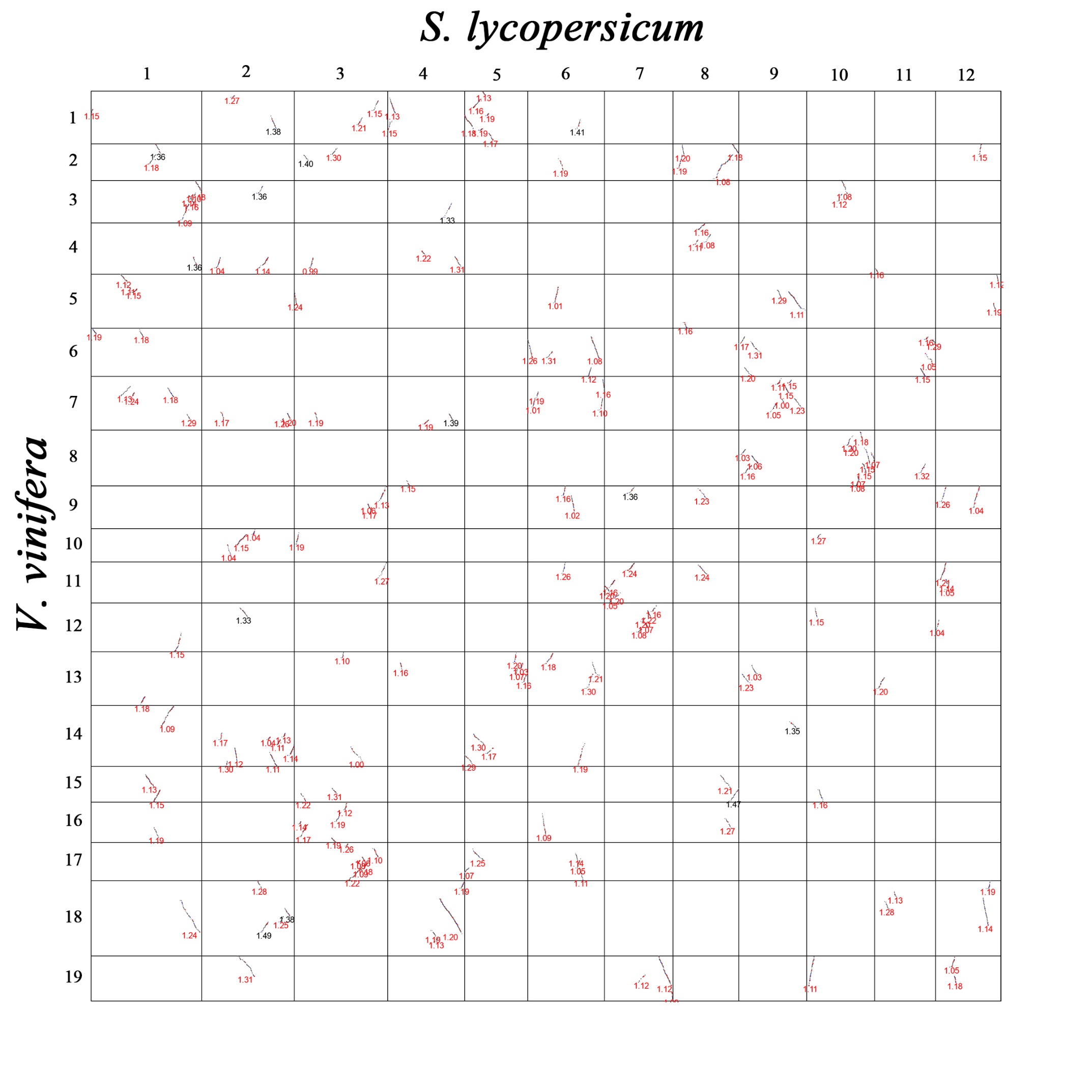


**Fig S2. *Ks* dot plot between *V. vinifera* and *S. lycopersicum* genome.**

Homologous synteny blocks dot plot between the genome of *V. vinifera* and *S. lycopersicum*, and the median *Ks* of blocks were marked, less than 1.325 is red, greater than 1.325 is black.


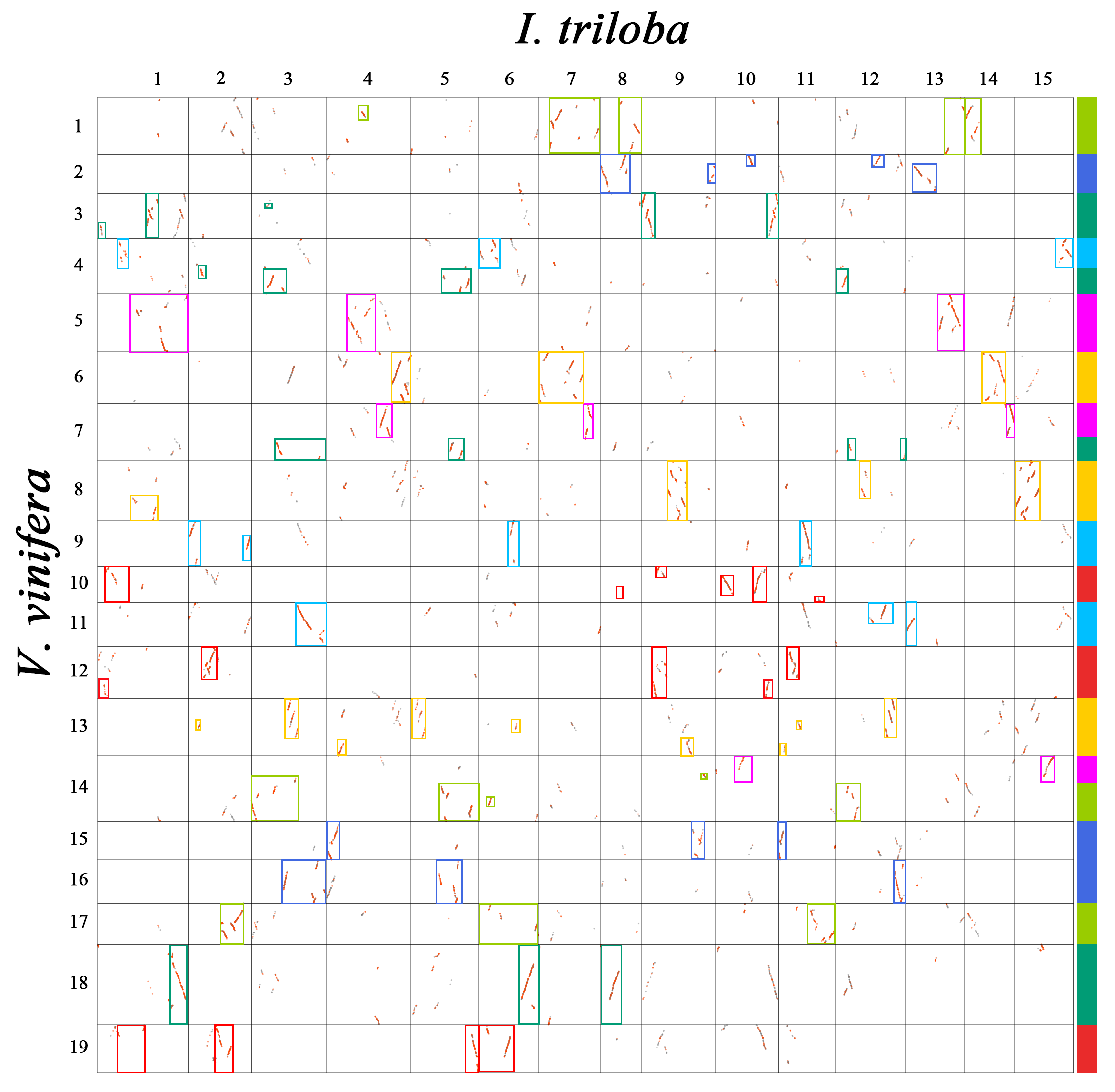


**Fig S3. Intergenomic homologous structure between the genome of *V. vinifera* and *I. triloba* genomes.**

The best, secondary, and other matched homologous gene pairs output by Blastp were plotted by red, gray, and gray colors in this figure, respectively. The dotplot shows only relatively large orthologous regions (containing more than 10 gene pairs). The highlighted box next to the *V. vinifera* chromosome corresponds to the seven colors of the eudicot’s ancestor, and the part with highlighted color in solid line frame is orthologous.


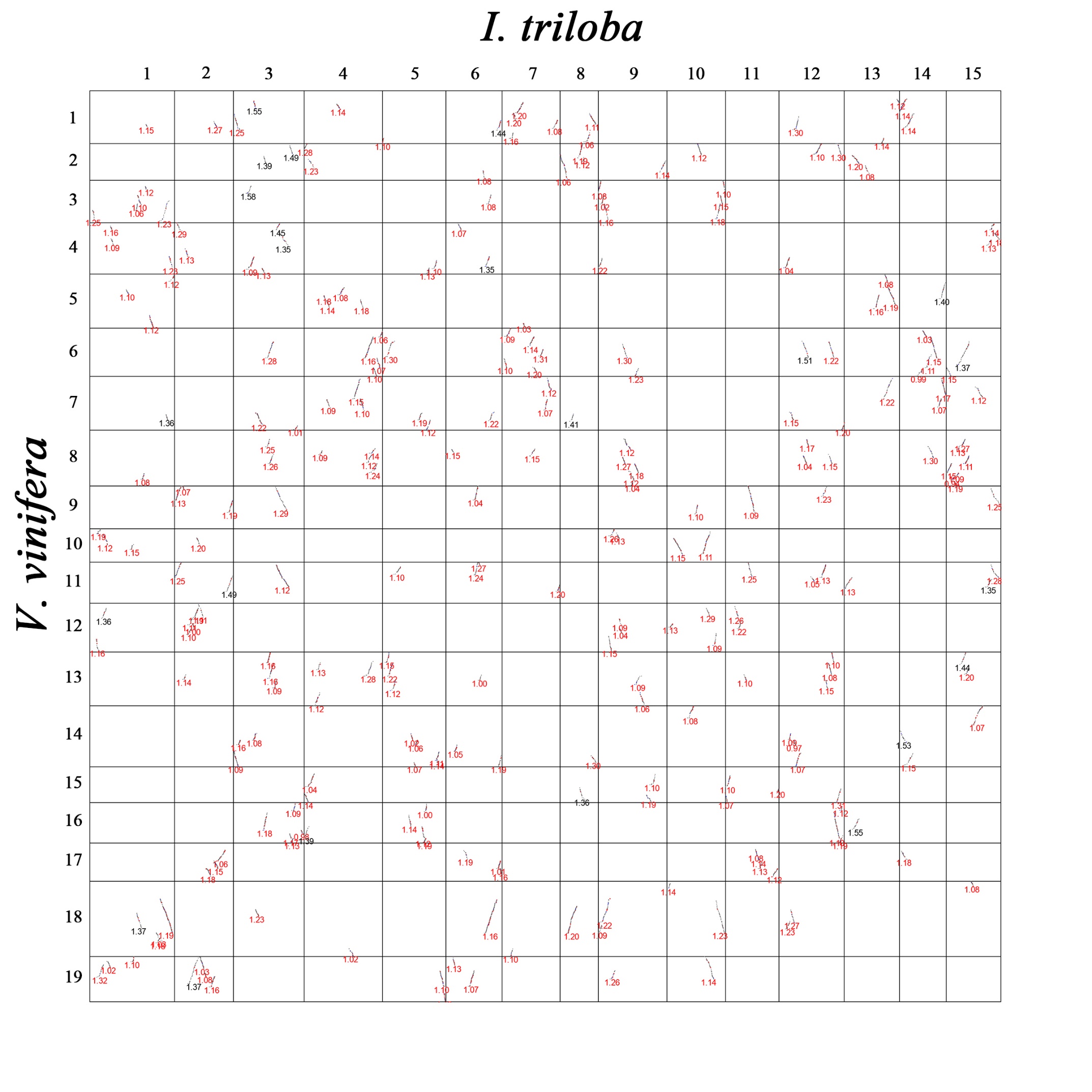


**Fig S4. *Ks* dot plot between *V. vinifera* and *I. triloba* genome.**

Homologous synteny blocks dot plot between the genome of *V. vinifera* and *I. triloba*, and the median *Ks* of blocks were marked, less than 1.325 is red, greater than 1.325 is black.


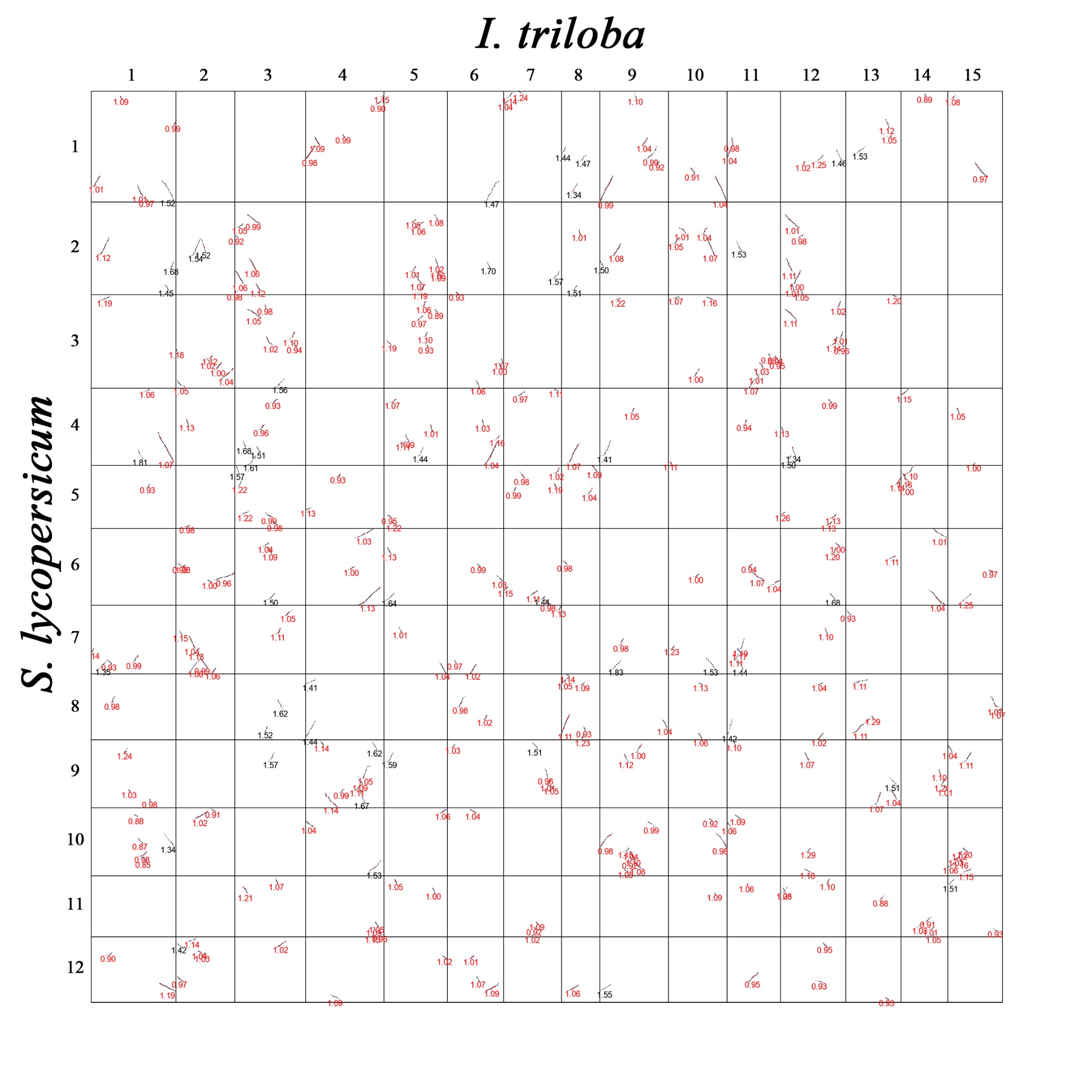


**Fig S5. Intergenomic homologous structure comparison analyses between** ***S. lycopersicum* and *I. triloba* genomes.**

The best, secondary, and other matched homologous gene pairs output by Blastp were plotted by red, gray, and gray colors in this figure, respectively. The dotplot shows only relatively large orthologous regions (containing more than 10 gene pairs). The part with highlighted color in solid line frame is orthologous.


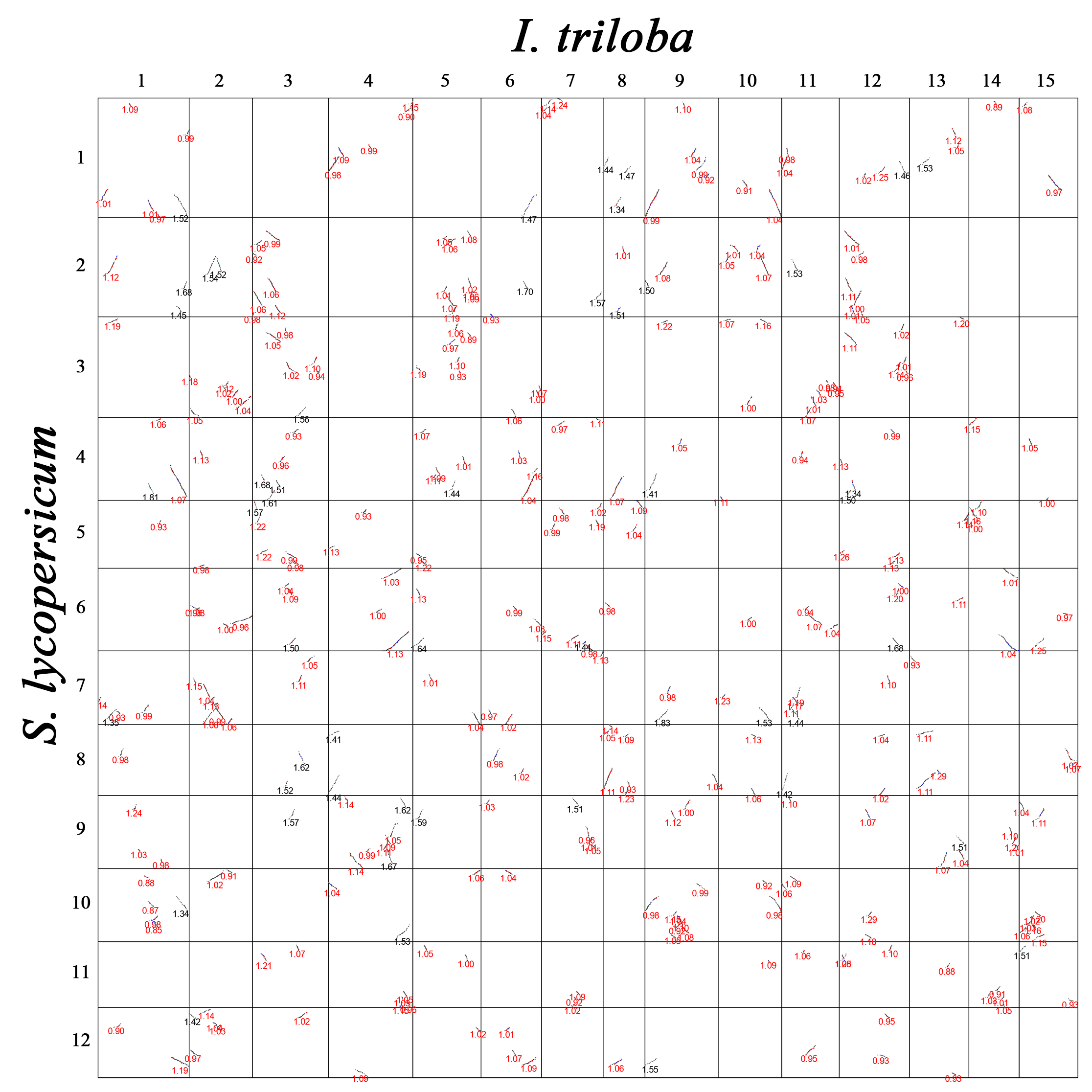


**Fig S6. *Ks* dot plot between *S. lycopersicum* and *I. triloba* genomes.**

Homologous synteny blocks dot plot between the genome of *S. lycopersicum* and *I. triloba* chinensis, and the median *Ks* of blocks were marked, less than 1.325 is red, greater than 1.325 is black.


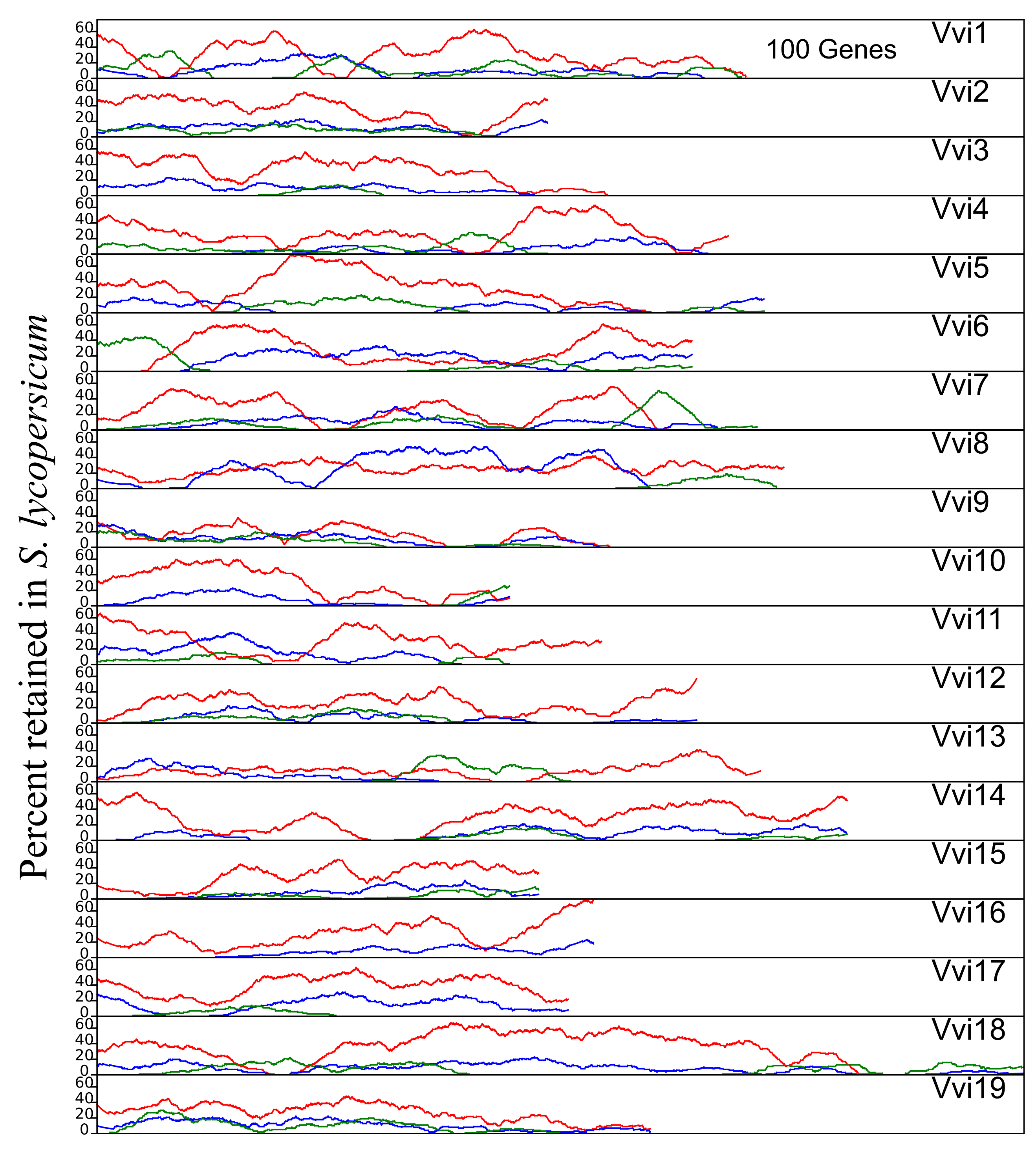


**Fig S7. *S. lycopersicum* gene retention along corresponding orthologous *V. vinifera* chromosome 1-19.**

Using the *V. vinifera* chromosomes as the reference, with 100 genes as a sliding window, the percentage of gene retention in the three *S. lycopersicum* subgenomes is shown in red, green and blue.


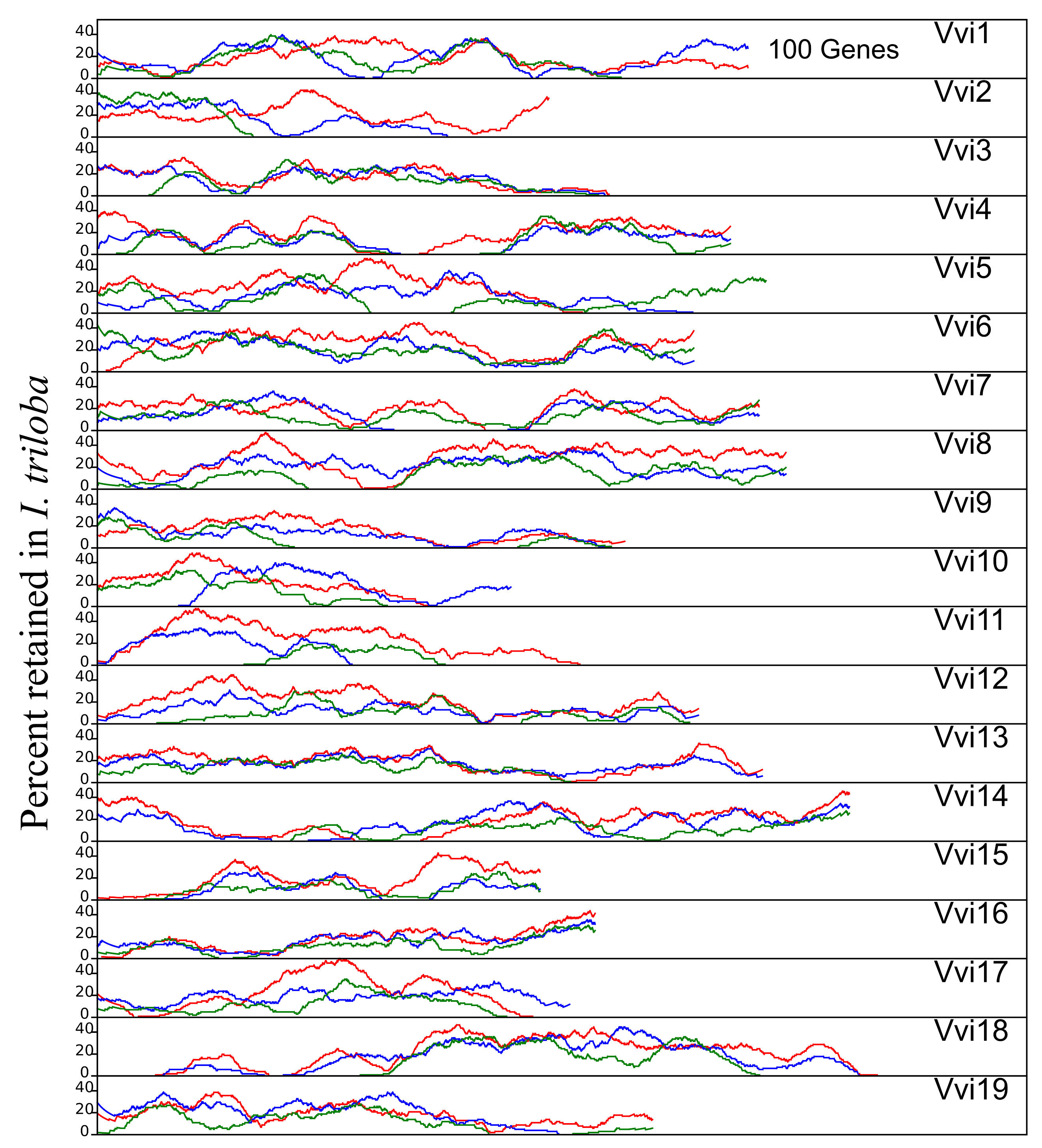


**Fig S8.** ***I. triloba* gene retention along corresponding orthologous *V. vinifera* chromosome 1-19.**

Using the *V. vinifera* chromosomes as the reference, with 100 genes as a sliding window, the percentage of gene retention in the three *I. triloba* subgenomes is shown in red, green and blue.

**
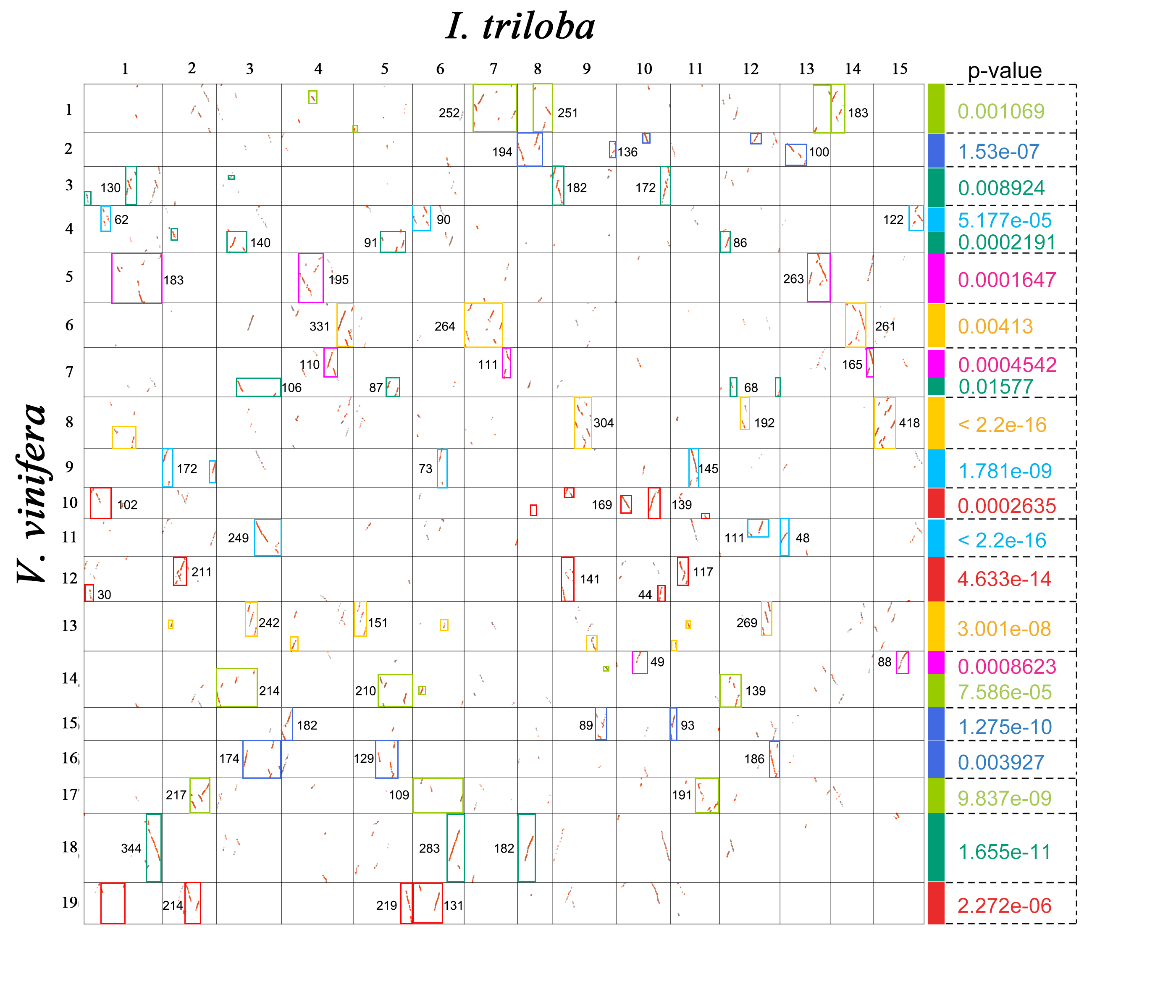
**

**Fig S9. Intergenomic homologous structure comparison analyses between *V. vinifera* and *I. triloba* genomes.**

The best, secondary, and other matched homologous gene pairs output by Blastp are plotted in red, gray, and gray respectively. The dotplot shows only relatively large orthologous regions (containing more than 10 gene pairs). The number of genes in these blocks regions is specified near the homologous gene region. The *P-value* describes whether the number of genes on the homologous fragment is significantly different in the three subgenomes. The *P-value* < 0.05 indicates that the significant difference in retention among the three subgenomes of *I. triloba* is credible, and then indicates that the three subgenomes are heterologous.


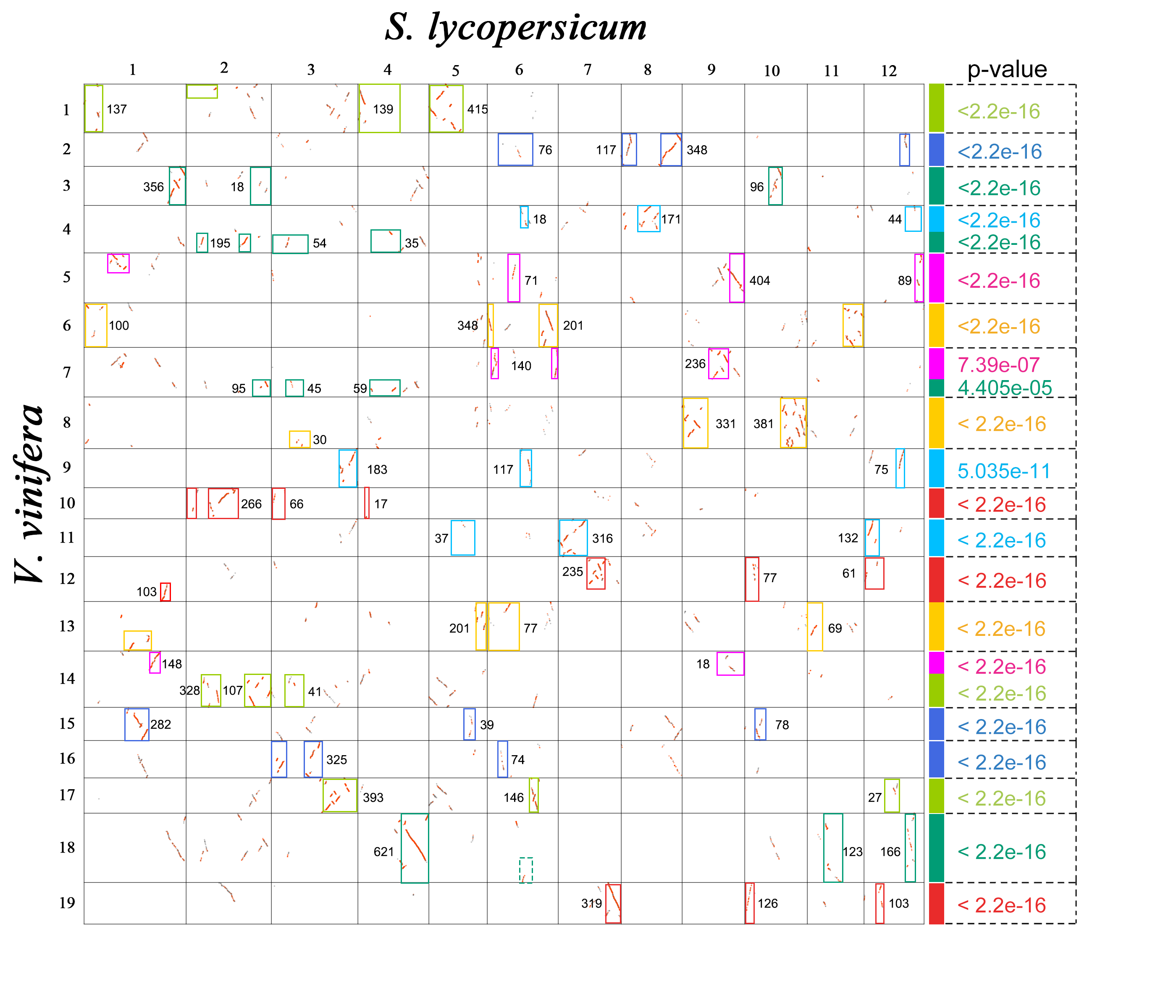


**Fig S10. Intergenomic homologous structure comparison analyses between *V. vinifera* and *S. lycopersicum* genomes.**

The best, secondary, and other matched homologous gene pairs output by Blastp are plotted in red, gray, and gray respectively. The dotplot shows only relatively large orthologous regions (containing more than 10 gene pairs). The number of genes in these blocks regions is specified near the homologous gene region. The *P-value* describes whether the number of genes on the homologous fragment is significantly different in the three subgenomes. The *P-value* < 0.05 indicates that the significant difference in retention among the three subgenomes of *S. lycopersicum* is credible, and then indicates that the three subgenomes are heterologous.


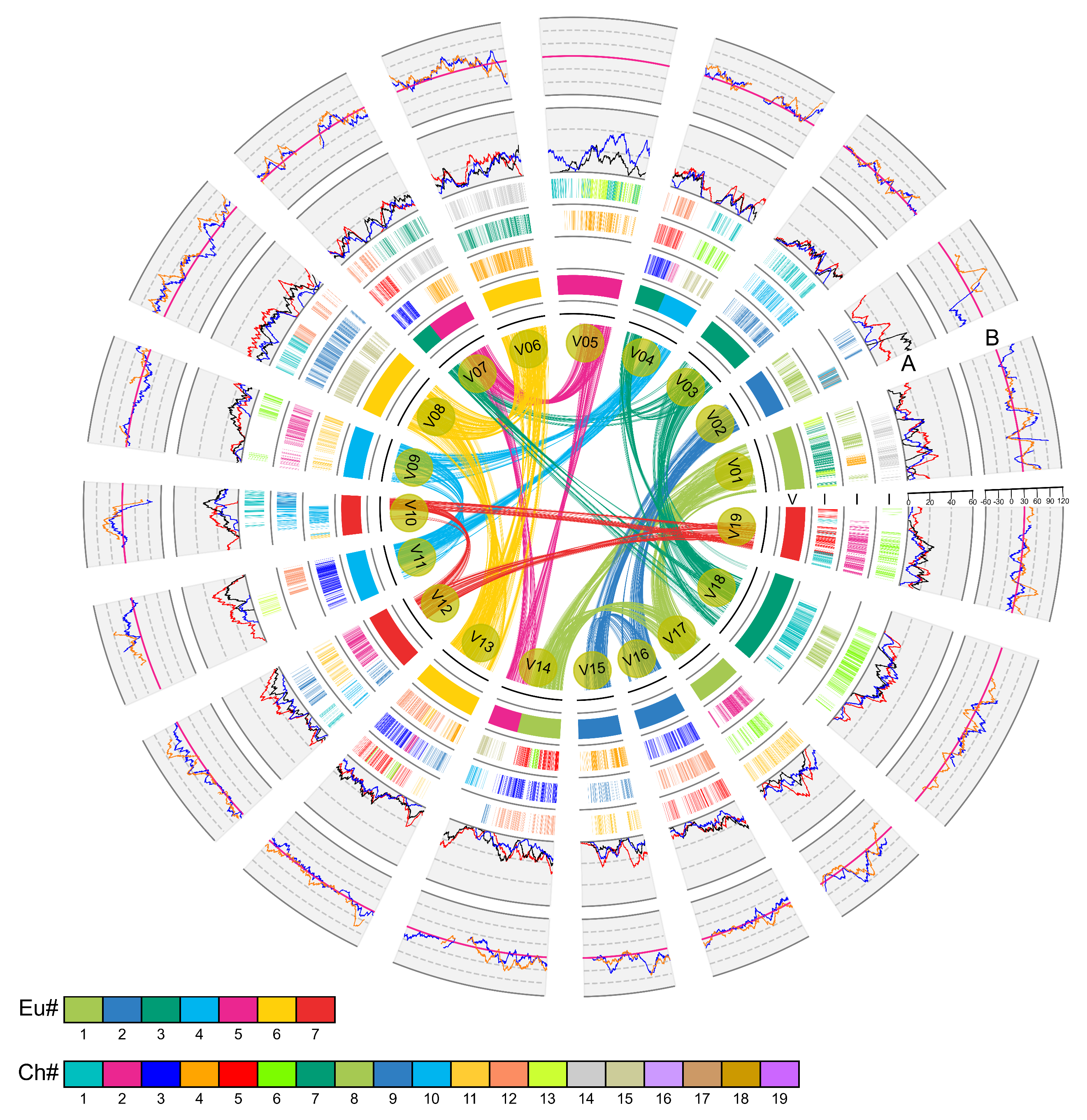


**Fig S11.** **Genomic alignments and gene retention of *I. triloba* sub-genomes along corresponding orthologous *V. vinifera* chromosomes.**

Genomic alignments and gene retention of *I. triloba* subgenomes along corresponding orthologous *V. vinifera* chromosomes. The genes in 19 chromosomes of *V. vinifera* within the inner circle, colored by the seven ancestral chromosomes of core eudicots as shown in the color scheme at the bottom (denoted by Eu#). Compared to the *V. vinifera* genome, the genomic paralogous and orthologous information within and among the genome of *I. triloba* in subgenomes is displayed in three circles. Each circle was formed by short vertical lines that denoted homologous orthologous genes, which were colored to indicate the *I. triloba* 12 chromosome numbers in their respective source plant, as shown in the color scheme at the bottom (denoted by Chr#). ①, Gene retention level of the least fractionated regions (LF, red), the medium fractionated regions (MF1) (blue), and the most fractionated regions (MF2) (black) in *I. triloba*. ②, The difference in gene retention between LF and MF1 (pink) and between LF and MF2 (blue).
